# Proceedings of the 18th Annual Conference of INEBRIA

**DOI:** 10.1186/s13722-023-00371-4

**Published:** 2023-04-13

## Oral presentations

### O1 Using pay-for-performance as a strategy to improve the consistency and quality of implementing a motivational interviewing-based brief intervention for people with HIV and substance use disorders

#### Bryan Garner^1^*, Stephen J. Tueller^2^, Michael Bradshaw^2^, Kathryn J. Speck^3^, Denna Vandersloot^4^, Mathew R. Roosa^5^, James Ford II^6^, Mark Zehner^6^, Jackie Mungo^2^, Sarah McDaniel^2^, Richa Ruwala^2^, Derek Satre^7^

##### ^1^The Ohio State University, Columbus, OH 43218-2646, USA; ^2^RTI International, Research Triangle Park, NC 27709, USA; ^3^The University of Nebraska, Lincoln, NE 68336, USA; ^4^Vandersloot Consulting, Portland, OR 97220, USA; ^5^Roosa Consulting, Syracuse, NY 13120, USA; ^6^University of Wisconsin-Madison, Madison, WI 53706, USA; ^7^University of California-San Francisco, San Francisco, CA 94143, USA

###### **Correspondence:** Bryan Garner (bryan.garner@osumc.edu)

*Addiction Science & Clinical Practice* 2023, **18(Suppl 1)**: O1

**Background:** As part of a dual-randomized type 2 implementation-effectiveness hybrid trial, which included 39 HIV service organizations (HSOs) across the U.S., 78 HSO staff, and 824 client participants with HIV and a substance use disorder (SUD), a motivational interviewing-based brief intervention (MIBI) was found to be effective. Importantly, the MIBI was only effective when implemented within the context of HSOs receiving the Implementation and Sustainment Facilitation (ISF) strategy (i.e., monthly 30–60 min team-focused facilitation meetings via Zoom) as an adjunct to the multifaceted control strategy (i.e., staff-focused training, feedback, and consultation regarding the MIBI), referred to as TFC. This presentation highlights results from the subsequent Substance Abuse Treatment to HIV Care II (SAT2HIV-II Project) ‚Äì a cluster-randomized type 3 implementation-effectiveness hybrid trial testing the effectiveness of pay-for-performance (P4P; TFC + ISF + P4P) to improve MIBI implementation beyond the TFC + ISF strategy.

**Materials and methods:** As part of the SAT2HIV-II Project (NCT04687917) approved by the Advarra IRB, 25 HSOs as well as participating staff and clients, were cluster randomized to either the control strategy (TFC + ISF) or the experimental strategy (TFC + ISF + P4P). MIBI staff working at HSOs randomized to the experimental strategy had the opportunity to receive $10 USD per MIBI implemented, as well as $10 USD per MIBI implemented at or above the 80th percentile level of fidelity. Guided by the Theory of Implementation Effectiveness, the primary implementation outcome measure was implementation effectiveness (i.e., the consistency and quality of MIBI implementation), a staff-level measure representing the standardized sum of the total number of MIBIs implemented and the total quality/fidelity scores.

**Results:** The P4P strategy had a medium-sized effect (d = 0.47) that significantly (p = 0.001) improved staff-level implementation effectiveness during the project‚Äôs implementation phase.

**Conclusions:** Consistent with prior research, the current research supports P4P as a highly-promising strategy to improve the implementation of brief motivational interventions.

### O2 Text-messaging brief interventions for hazardous drinking and tobacco use intervention development lessons from India with digital alcohol interventions

#### Abhijit Nadkarni^1*^

##### ^1^Department of Population Health, London School of Hygiene & Tropical Medicine, London, WC1E 7HT, UK

###### **Correspondence:** Abhijit Nadkarni (abhijit.nadkarni@lshtm.ac.uk)

*Addiction Science & Clinical Practice* 2023, **18(Suppl 1)**: O2

**Background:** Despite the large and increasing burden of hazardous drinking and tobacco use in India, access to appropriate interventions remains limited because of human resource shortages. The aim of AMBIT and ToQuit was to develop contextually appropriate text-messaging brief interventions (BIs) for hazardous drinking and tobacco use respectively using a systematic and evidence based intervention development process.

**Methods:** The intervention development process included (a) examination and synthesis of global evidence on effectiveness of text messaging interventions for the target conditions; (b) in-depth qualitative interviews with a range of stakeholders such as hazardous drinkers, tobacco users, and health professionals; (c) Delphi surveys to refine intervention content; (d) intervention development workshops; (e) intervention cohorts with measurement of process data and pre- post- outcome evaluation to evaluate acceptability and feasibility of the interventions; and (f) pilot RCTs with nested qualitative studies to examine feasibility of trial procedures.

**Results:** Both the interventions resulted from the triangulation of outputs from the participatory methods described above. The stepped intervention development process resulted in iterative changes made to the interventions to make them contextually relevant, thus enhancing the feasibility and acceptability. Both interventions were acceptable to the target groups and feasible to be delivered through text messaging. Pilot RCT results indicated a trend in favour of the BIs when compared to the control arm.

**Conclusion:** Rigorous and systematic intervention development processes are important to develop contextually relevant interventions. If demonstrated to be cost-effective in definitive trials, ToQuit and AMBIT have the potential to reduce the treatment gaps for hazardous drinking and tobacco use through increasing access to care.

### O3 Bot or not? Pitfalls and good practices in data management in digital alcohol research

#### Gemma Loebenberg^1^, Larisa Dinu^1^

##### ^1^UCL Tobacco and Alcohol Research Group, Department of Behavioural Science and Health, University College London, London, WC1E 7HB, UK

###### **Correspondence:** Gemma Loebenberg (gemma.loebenberg@ucl.ac.uk)

*Addiction Science & Clinical Practice* 2023, **18(Suppl 1)**: O3

**Background:** Digital alcohol research is popular due to lower costs, and the relative convenience of recruiting large numbers of participants compared with in-person research. However, digital participation with little verification is open to automated “bots” and manual fraud. This paper aims to highlight issues with fraudulent entries affecting digital alcohol research with financial incentives, and the importance of rigorous data management to identify and rectify problems.

**Materials and methods:** A randomised controlled trial (n = 5562) evaluating the effectiveness of an alcohol reduction app, Drink Less, recruited participants online from July 2020-March 2022. Follow-up occurred at three timepoints with financial incentives available. Numerous “bots”—automated responses generated in clusters—were identified during the first month’s recruitment. Manual fraud—participants providing false information or duplicate entries—was detected thereafter. Both were initially identified by address authentication, then telephone verification.

**Results:** 863 participants (75.6%) were identified as bots during data screening between 13th July-12th September 2020. A Captcha was added to the survey on 11th August 2020; no new bots were identified as signing up after this. Ongoing checks throughout the study detected additional suspicious responses caused by people engaging in manual fraud, particularly during November 2020, with 110 (34%) entries identified as fraudulent, and October-December 2021 with 112 (22.4%). Further screening questions, removing the prominence of financial incentives from social media advertising, and an additional requirement to provide a mobile phone number for verification reduced those detected engaging in manual fraud to a negligible level with only 1 (0.16%) identified in February 2022.

**Conclusions:** Data management protocols are necessary to detect and minimise automated bots and manual fraud in digital alcohol trials. When creating online surveys, researchers should use fraud detection software offered (e.g., Captchas), consider how to advertise the study, identify and deter duplicate entries, and avoid prominence of financial incentives.

### O4 Methodological insights from a remote Randomised Controlled Trial examining the effectiveness of an alcohol reduction app in helping increasing and higher risk drinkers reduce their alcohol consumption

#### Melissa Oldham^1*^

##### ^1^UCL Tobacco and Alcohol Research Group, Department of Behavioural Science and Health, University College London, London, WC1E 7HB, UK

###### **Correspondence:** Melissa Oldham (m.oldham@ucl.ac.uk)

*Addiction Science & Clinical Practice* 2023, **18(Suppl 1)**: O4

**Background:** Randomised Controlled Trials (RCTs) with no in-person contact between researchers and participants present unique challenges. We present methodological insights from a large, remote RCT examining the effectiveness of an alcohol reduction app. We highlight how different sources of remote recruitment compare in terms of cost-effectiveness, retention rates, data quality and demographic diversity. We also outline the costs associated with different follow-up methods and outline broader challenges and recommendations around data quality.

**Materials and methods:** A blinded descriptive analysis of 5607 hazardous and harmful drinkers (Alcohol Use Disorders Identification Test (AUDIT) score ≥ 8), taking part in a two-arm, parallel group, remote RCT with a 1:1 allocation comparing the intervention (Drink Less) with usual digital care (NHS alcohol advice webpage). Participants were recruited using varying methods between July 2020 and March 2022. We evaluate the success and cost-effectiveness of different forms of remote recruitment (e.g. NHS website, social media and radio advertising) and different follow-up methods (emails, phone calls and postal surveys).

**Results:** The most cost-effective recruitment strategies were the free ones; the NHS advert (n = 1959) and advertising through local health providers (n = 15), but these had varying levels of success. Social media (£3.46 per person recruited) was more cost-effective than emailing users of the Smoke Free app (£6.81 pp) and radio advertising (£11.72 pp) but produced a less representative sample and higher levels of participant fraud. At 6-month-follow-up, the majority of participants responded after four emails (67%), with a further 10% responding after phone calls and 7% responding to postal surveys. Broader methodological recommendations for future remote trials are discussed including the importance of data checks and providing technical support for participants in accessing and using digital interventions.

**Conclusions:** We offer recommendations for cost effective strategies for remote recruitment and follow-up retention rates as well as broader recommendations to ensure data quality.

### O5 The effect of question order on outcomes in the core outcome set for brief alcohol interventions among online help-seekers

#### Marcus Bendtsen^1*^

##### ^1^Department of Health, Medicine and Caring sciences, Linköping University, Linköping, SE-581 83, Sweden

###### **Correspondence:** Marcus Bendtsen (marcus.bendtsen@liu.se)

*Addiction Science & Clinical Practice* 2023, **18(Suppl 1)**: O5

**Background:** A core outcome set (COS) has been developed through international consensus to reduce selective reporting and research waste, and guide outcome choice in brief alcohol intervention research. This study aimed to estimate order effects among questions in the COS.

**Methods:** Adults, aged over 18 years who searched online for alcohol-related help were invited to complete a survey containing the COS items. The order of four item clusters was randomised following a factorial design. Primary outcomes were order effects among the COS items and patterns of abandonment of the questionnaire.

**Results:** We randomised 7334 participants, of which 5256 had responded to at least one question and were available for primary and sensitivity analyses. Current non-drinkers were excluded. Median completion time for the COS was 4 min 16 s. We found evidence of order effects among COS clusters, including higher self-reported average consumption and odds of harmful and hazardous drinking among those who first answered questions on recent consumption and impact of alcohol use. Lower self-reported recent consumption was found among those first asked about average consumption. Quality of life was reported as lower among those who first responded to questions on impact of alcohol use, which in turn was lower among those who first answered question on average consumption and quality of life. Attrition was lowest when average consumption was asked first, and highest when quality of life or impact of alcohol use asked first.

**Conclusions:** Researchers designing studies should note that question order effects may exist. At a minimum, all study participants should be asked the same questions in the same order. There is no perfect question order; rather, researchers should be guided by the nature of the studied population, recruitment, additional questions, concerns about under-reporting, screening for inclusion, and retention concerns.

### O6 Smartphone-based secondary prevention intervention for university students with unhealthy alcohol use: a randomized controlled trial

#### Nicolas Bertholet^1^, Elodie Schmutz^1^, Joseph Studer^1^, Angéline Adam^1^, Gerhard Gmel^1^, John A. Cunningham^2,3,4^, Jennifer McNeely^5^, Jean-Bernard Daeppen^1^

##### ^1^Department of Psychiatry, Addiction Medicine, Lausanne University Hospital and Lausanne University, Lausanne, Switzerland; ^2^National Addiction Centre, Institute of Psychiatry, Psychology and Neuroscience, Kings College London, London, SE5 8BB, UK; ^3^Centre for Addiction and Mental Health, Institute of Mental Health and Policy Research, Toronto, Canada; ^4^Departments of Psychiatry and Psychology, University of Toronto, Toronto, Canada; ^5^Department of Population Health, New York University Grossman School of Medicine, New York, USA

###### **Correspondence:** Nicolas Bertholet (nicolas.bertholet@chuv.ch)

*Addiction Science & Clinical Practice* 2023, **18(Suppl 1)**: O6

**Background:** Unhealthy alcohol use is a leading cause of morbidity and mortality among university students. Smartphone-based interventions have the potential to reach large parts of the student population. We developed a proactive smartphone-based intervention for unhealthy alcohol use with the involvement of students and tested its efficacy in a randomized controlled trial.

**Methods:** 1770 students with unhealthy alcohol use identified by screening from four Swiss higher education institutions were randomized to receive access to a smartphone-based intervention (i.e. smartphone application) (n = 884) or to a no-intervention control condition (n = 886). Follow-ups were at 3 and 6 months. Primary outcome was number of standard drinks per week (SDW) at 6 months. Secondary outcome was number of heavy drinking days (HDD; past 30 days) at 6 months. The intervention effect on SDW and HDD were tested using negative binomial generalized linear mixed models with participants and recruitment sites as random effects and intervention and time as fixed effects (with an intervention by time interaction term). Models were adjusted for age and gender.

**Results:** Mean (SD) age was 22.34 (3.07), 54.1% were female; 66.0% were Bachelor students, 30.1% Master, 2.4% PhD, and 1.4% other. Baseline mean number of drinks per week was 8.60(8.17). Baseline number of HDD, past 30 days was 3.53 (4.02). Follow-up rate was 96.4% and 95.9% at 3 and 6 months, respectively. Compared to the no-intervention condition, those who received access to the intervention reported significantly fewer SDW (intervention by 3-month follow-up interaction, IRR [95%CI] 0.88 [0.82;0.96]; intervention by 6-month follow-up interaction, 0.88 [0.81;0.95]) and significantly fewer HDD (intervention by 3-month follow-up interaction, IRR 0.90 [0.81;0.99]; intervention by 6-month follow-up interaction, 0.88 [0.80;0.98]).

**Conclusion:** Providing access to a smartphone-based secondary prevention intervention was efficacious to reduce unhealthy alcohol use among university students. The intervention has the potential for widespread implementation.

### O7 Implementation and workflow strategies for integrating digital therapeutics for alcohol use disorders into primary care: a qualitative study

#### Jessica M. Mogk^1*^, Theresa E. Matson^1^, Ryan M. Caldeiro^2^, Angela M. Garza Mcwethy^2^, Tara Beatty^1^, Brandie C. Sevey^1^, Joseph E. Glass^1^

##### ^1^Kaiser Permanente Washington Health Research Institute, Seattle, WA 98101, USA; ^2^Kaiser Permanente Washington Mental Health & Wellness Services, PO Box 9010, Renton, WA 98057-9010, USA

###### **Correspondence:** Jessica M. Mogk (Jessica.M.Mogk@kp.org)

*Addiction Science & Clinical Practice* 2023, **18(Suppl 1)**: O7

**Background:** Alcohol use disorders (AUD) are prevalent and often go untreated. Patients are commonly screened for AUD in primary care, but existing treatment programs are failing to meet demand. Digital therapeutics are a cost-effective treatment option that may help fill treatment gaps. The goal of this study was to identify implementation needs and workflow design considerations for integrating digital therapeutics for AUD into primary care.

**Materials and methods:** We conducted qualitative interviews with clinicians, care delivery leaders, and quality improvement staff (n = 16) in an integrated healthcare delivery system in the United States. All participants had experience implementing digital therapeutics for depression and substance use disorders in primary care. Interviews were designed to gain insights into adaptations needed to optimize existing clinical processes, workflows, and implementation strategies for use with alcohol-focused digital therapeutics. Interviews were recorded and transcribed and then analyzed using a rapid analysis process and affinity diagramming.

**Results:** Participants were enthusiastic about digital therapeutics for AUD and anticipated high patient demand for such a resource. While many participants expressed confidence that previous implementation strategies would be effective for the implementation of digital therapeutics for AUD, they suggested adaptations to accommodate high patient volume and to support patients with varying AUD severity. Several participants advocated for simplified workflows (e.g., instead of having to go through a provider, patients could connect to the app directly). Participants hypothesized that patients who are self-motivated to reduce drinking would be best suited for digital therapeutics; they also recommended that apps include goals besides abstinence.

**Conclusions:** The implementation of digital therapeutics for AUD would benefit from careful consideration of the target population. Optimal integration requires tailoring workflows to meet anticipated patient volume and designing workflow and implementation strategies to meet the unique needs of patients with varying AUD severity.

### O8 Digital therapeutics for opioids and other substance use disorders (DIGITS Trial) in primary care: results of a quality improvement pilot and study protocol for a hybrid type-III implementation trial

#### Joseph E. Glass^1^, Tara Beatty^1^, Angela M. Garza Mcwethy^2^, Edwin S. Wong^3^, Ryan M. Caldeiro^2^, Abisola Idu^1^, Jennifer F. Bobb^1^, Jessica M. Mogk^4^, Kelsey L Stefanik-Guizlo^4^, Dustin Key^1^, Caitlin N. Dorsey^1^, Lorella Palazzo^1^, Katharine A. Bradley^1^

##### ^1^Kaiser Permanente Washington Health Research Institute, Seattle, WA 98101-1466, USA; ^2^Kaiser Permanente Washington Mental Health & Wellness Services, PO Box 9010, Renton, WA 98057-9010, USA; ^3^Department of Health Services, University of Washington, Box 357660, Seattle, WA 98195, USA; ^4^Center for Accelerating Care Transformation, Kaiser Permanente Washington Health Research Institute, Seattle, WA 98101, USA

###### **Correspondence:** Joseph E. Glass (joseph.e.glass@kp.org)

*Addiction Science & Clinical Practice* 2023, **18(Suppl 1)**: O8

**Background/Objectives:** reSET® and reSET-O® are the first FDA-authorized prescription digital therapeutics (PDTs) for substance use disorders and opioid use disorder, respectively. More information is needed on how to optimally engage primary care clinicians and patients in using these treatments, which include the community reinforcement approach and contingency management. Objectives: (1) Describe results of a pilot of reSET/reSET-O implementation in primary care. (2) Describe the protocol for the DIGITS Trial, a cluster-randomized, hybrid type-III implementation trial testing clinic- and patient-level strategies for implementing reSET/reSET-O in primary care.

**Methods:** From February to May 2021, researchers partnered with Kaiser Permanente Washington clinical leaders on a quality improvement pilot of reSET/reSET-O involving two clinicians in two primary care clinics. Goals were to iteratively refine clinical workflows and a standard implementation strategy. In December 2021, the DIGITS Trial launched, where primary care sites (currently n = 19) use standard implementation strategies such as clinician training and performance monitoring. Clinics are randomized in a 2 × 2 factorial design to receive up to two added implementation strategies: (1) external practice facilitation, a clinic-level implementation strategy, and/or (2) health coaching, a patient-level implementation strategy. Primary outcomes include reach (proportion of patients prescribed reSET/reSET-O) and fidelity (patient engagement with reSET/reSET-O). Secondary outcomes include substance use and health care costs.

**Results:** In the 12-week quality improvement pilot, clinicians prescribed reSET or reSET-O to 13 patients and 8 patients activated their prescription. Improvements were made to workflows, training materials, EHR tools, and data management procedures to be leveraged in the trial.

**Conclusions:** Offering digital therapeutics for substance use disorders, including opioids, in primary care appears feasible. The DIGITS Trial will provide data on the potential benefits and cost-effectiveness of implementation strategies.

### O9 A proposed framework for designing trials, evaluating the effectiveness and implementation of digital interventions for substance use

#### Theresa E. Matson^1*^, Eric D.A. Hermes^2^, Aaron R. Lyon^3^, Andrew Quanbeck^4^, Stephen M. Schueller^5^, Sarah (“Sadie”) M. Wilson^6^, Joseph E. Glass^1^

##### ^1^Kaiser Permanente Washington Health Research Institute, Seattle WA 98101-1466, USA; ^2^School of Medicine, Yale University, New Haven CT 06510, USA; ^3^Department of Psychiatry and Behavioral Sciences, School of Medicine, University of Washington, Seattle WA, USA; ^4^Department of Family Medicine and Community Health, University of Wisconsin-Madison, Madison WI 53075, USA; ^5^School of Social Ecology, University of California, Irvine, Irvine CA 92697, USA; ^6^Department of Psychiatry & Behavioral Sciences, Duke University School of Medicine, Durham NC 27710, USA; Center of Innovation to Accelerate Discovery and Practice Transformation, Durham VA Health Care System, Durham NC 27705, USA

###### **Correspondence:** Theresa E. Matson (tessa.e.matson@kp.org)

*Addiction Science & Clinical Practice* 2023, **18(Suppl 1)**: O9

**Background:** Clinicians and community health workers may wish to use digital interventions to reach more patients with unhealthy substance use, optimize costs of care, and improve outcomes. However, digital interventions have unique implementation considerations (e.g., technology infrastructure, digital literacy, monitoring and follow-up) and may not fit traditional care pathways. Effectiveness and implementation trials are needed to understand how well digital interventions work and how to best deploy them in the real-world. This presentation presents a framework to help researchers design their trials in such a way that maximizes scientific understanding.

**Methods:** This framework draws from the literature on trial design, expert perspectives on the use of digital interventions, and lessons learned from implementation science research programs. It outlines three major steps for designing trials of digital interventions: (1) framing the research question; (2) delineating components of the intervention, implementation strategy, and delivery approach; and (3) specifying the experiment and other elements of trial design.

**Results:** In Step 1 of this framework, researchers frame the research question in terms of the goals or activities to be tested (i.e., features of the digital intervention itself, specific implementation strategies, or level of clinical support). In Step 2, researchers define and delineate each study component as actor, activity, action target, or outcome to maximize inference and reproducibility across studies. Steps 1 and 2 inform Step 3, in which researchers specify features of the trial design (i.e., experimental/comparator selection, outcome selection, and design classification). To illustrate the utility of this framework, we compare and contrast implementation and effectiveness studies of digital interventions for substance use.

**Conclusions:** The proposed framework provides a foundation for designing trials of digital interventions for substance use in healthcare and community settings. This framework can help researchers decide on appropriate methodology and help decision-makers understand how to apply findings.

### O10 A two-arm parallel-group individually randomised prison pilot study of a male remand alcohol brief intervention for self efficacy enhancement: the APPRAISE study

#### Gillian Waller^1*^, Jennifer Ferguson^1^, Aisha Holloway^2^, Jamie B Smith^2^, Dorothy Newbury-Birch^3^

##### ^1^School of Social Sciences, Humanities and Law, Teesside University, Middlesbrough, TS1 3BX, UK; ^2^School of Health in Social Science, University of Edinburgh, Edinburgh, EH8 9AG, UK; ^3^SSSHL Department of Humanities and Social Sciences, Teesside University, TS1 3BX, USA

###### **Correspondence:** Gillian Waller (gillian.waller@outlook.com)

*Addiction Science & Clinical Practice* 2023, **18(Suppl 1)**: O10

**Background:** The prevalence of at-risk drinking is higher amongst individuals within the criminal justice system, with 51–83% of incarcerated people being classified as risky drinkers [1] and for individuals on remand in prison, the prevalence of risky drinking is consistently higher (62–68%) [2].

**Materials and methods:** APPRAISE is a two-arm, parallel group, individually randomised, pilot study of an alcohol brief intervention (ABI), which was developed for use with men on remand, across two prison sites in North-East England and Scotland [3]. Semi-structured interviews were used to explore the acceptability and feasibility of the ABI with (i) male remand prisoners who had received the in-prison ABI, (ii) interventionists who had delivered the ABI, and (iii) stakeholders involved with the running or the management of the pilot.

**Results:** Findings confirmed that in-prison ABI sessions were more acceptable and feasible than post-release sessions, due to being unable to reestablish contact and issues with relapse upon release. Findings from the prisoner interviews suggested that males welcomed receiving support with making changes to their alcohol consumption and preferred continuous support. It was important for males to feel comfortable and trusting of an interventionist before sharing their personal experiences and habits. Findings from the interventionists were that the prison setting and culture affected the acceptability and the feasibility of the ABI, due to the lack of available space and staff capacity. The ABI training and content appeared to be acceptable, although the length and content could be condensed. The ABI delivery benefited from staff buying into the intervention and being motivated and engaged. The stakeholder data identified the importance of engagement with prisoners and the lack of currently available support for males on remand.

**Conclusion:** The APPRAISE ABI appeared to be feasible and acceptable to implement, although future work should focus on determining the feasibility and acceptability of the ABI on a wider scale.

### O11 An exploration of delivering screening and brief interventions for women leaving prison, a holistic approach

#### Jennifer Ferguson^1*^, Dorothy Newbury-Birch^2^, Aisha Holloway^3^

##### ^1^School of Social Sciences, Humanities and Law, Teesside University, Middlesbrough, TS1 3BX, UK; ^2^SSSHL Department of Humanities and Social Sciences, Teesside University, TS1 3BX, UK; ^3^School of Health in Social Science, University of Edinburgh, Edinburgh, EH8 9AG, UK

###### **Correspondence:** Jennifer Ferguson (jennifer.ferguson@tees.ac.uk)

*Addiction Science & Clinical Practice* 2023, **18(Suppl 1)**: O11

**Background:** Whilst it is well evidenced that the prevalence of alcohol misuse is high in the criminal justice system, and it can be shown it is for women on their own also, it is important to investigate the differences between men and women, in order to tailor interventions to this specific population. More females are found to be risky drinkers when they arrive in prison (24%) compared to males (18%) and there are established gendered pains of imprisonment. Early PhD work by JF explored this unmet need, this work further explores this with a particular focus on prison staff delivering the intervention.

**Materials and methods:** Two systematic reviews and 18 in depth qualitative interviews were carried out with women in an open prison and relevant staff and stakeholders. Thematic analysis of the transcripts was undertaken and triangulated with the reviews and recommendations for a future pilot study were made.

**Results:** The findings of the qualitative work aligned with the already evidenced pains of imprisonment. The research highlighted the importance of using the 10 question AUDIT to establish rapport as well as its main purpose of screening. However, whilst participants discussed pragmatic issues but one of the main findings was the element of staff rapport within the setting. It was surprisingly a uniformed officer who was most favoured for delivery of the intervention.

**Conclusions:** SBI with women in an open prison setting is both feasible and acceptable but the importance is around who delivers the SBI, with existing staff being favourable to the women.

### O12 Examining the role of vulnerabilities on alcohol consumption in the age of COVID19 and their implication for interventions

#### Andrew Divers^1*^, Judith Eberhardt^2^, Dorothy Newbury-Birch^3^

##### ^1^School of Social Sciences, Humanities and Law, Teesside University, Middlesbrough, TS1 3BX, UK; ^2^SSHL Psychology Centre for Applied Psychological Science, Teesside University, Middlesbrough, TS1 3BX, UK; ^3^SSSHL Department of Humanities and Social Sciences, Teesside University, Middlesbrough, , TS1 3BX, UK

###### **Correspondence:** Andrew Divers (a.divers@tees.ac.uk)

*Addiction Science & Clinical Practice* 2023, **18(Suppl 1)**: O12

**Background:** Understanding the factors that may lead to alcohol misuse and problematic drinking is essential to understanding how best to design interventions to ameliorate this. Evidence suggests that in the UK, alcohol consumption has increased dramatically within certain regions and populations, with those households purchasing the most alcohol increasing these excess purchases 17 times more than those purchasing the least (1) during the COVID-19 pandemic. These increases, alongside the deepening of existing inequalities and creation of new pressures during COVID-19 suggests a need reassess these factors in light of the pandemic.

**Materials and methods:** To date 1242 responses to online questionnaires have been gathered as well as the completion of 106 semi-structured interviews. Data from these are presented, having undergone thematic analysis and their implication for future interventions are discussed.

**Results:** The self-reported impact of the pandemic, particularly lockdowns on the general wellbeing of participants was significant, although not always negative. However for those that indicated that they did suffer a reduction in wellbeing (and increase in alcohol consumption) sometimes multiple vulnerabilities were also implicated.

**Conclusions:** Vulnerability can take many forms—cultural, economic, and social being just some of these. Interventions targeting problematic alcohol consumption in communities must be aware of these vulnerabilities and the role they play in order to ensure their impact, particularly in unprecedented times.

### O13 Safe and Wellbeing Visits: a brief intervention tool to drive forward population health improvements using the fire service in County Durham

#### Natalie Connor^1*^, Mark Henderson^2^, Rob Cherrie^2^, Gill O'Neill^3^, Dorothy Newbury-Birch^4^

##### ^1^School of Social Sciences, Humanities and Law, Teesside University, Middlesbrough, TS1 3BX, UK; ^2^County Durham and Darlington Fire & Rescue Service; ^3^Durham County Council^4^SSSHL Department of Humanities and Social Sciences, Teesside University, Middlesbrough, TS1 3BX, UK

###### **Correspondence:** Natalie Connor (n.connor@tees.ac.uk)

*Addiction Science & Clinical Practice* 2023, **18(Suppl 1)**: O13

**Background:** Reductions in dwelling fires are largely due to prevention work carried out by the Fire and Rescue Service (FRS). Safe and Wellbeing Visits (SWV) were introduced in 2016 by County Durham and Darlington Fire and Rescue Service focusing on preventative health as well as fire. Prevention work carried out by the FRS is enabled by its reputation as a trusted organisation. Vulnerable residents at risk from fire as well as a range of health conditions are an ideal audience for the FRS to target brief interventions. One of the health conditions screened for in the SWV is risky alcohol consumption, with it being a risk factor for dwelling fires.

**Materials and methods:** A mixed methods approach was used to analyse SWV referral data, alongside focus groups (N = 7) and interviews (N = 16) with service beneficiaries, delivery staff and partners.

**Results:** 11,485 individuals were screened for risky alcohol consumption over a two year period. 0.4% were eligible for referral. There were high levels of engagement to services for dementia, loneliness and isolation, and a Warm Home scheme. Lower levels of engagement were experienced for alcohol, smoking and slips, trips and falls. Qualitative analysis allowed key strategic and operational themes to be developed.

**Conclusion:** Although SWVs provide an opportunity for early intervention with those with risky alcohol consumption, less than 1% are identified. In addition, services are then experiencing low levels of engagement. Further work is required to explore this, as other referrals from SWVs are experiencing higher levels of identification and engagement.

### O14 Screening for and assessing alcohol use and consequences in adolescents

#### Paul Toner^1*^, Jim McCambridge^2^, Jan R. Böhnke^3^

##### ^1^Queen's University Belfast, Belfast, UK; ^2^University of York, York, UK; ^3^University of Dundee, Dundee, UK

###### **Correspondence:** Paul Toner (p.toner@qub.ac.uk)

*Addiction Science & Clinical Practice* 2023, **18(Suppl 1)**: O14

**Aims:** This programme of work was stimulated by a gap in knowledge identified in research, policy and practice literature and aimed to develop an item bank to screen for and assess the continuum of alcohol risk and harm in adolescents.

**Methods:** The project adopted a sequential mixed methods design integrating thematic and advanced psychometric analyses. Semi-structured interviews with 44 adolescents in a range of UK settings including: schools, supported accommodation, criminal justice settings, community groups; were conducted to develop and refine item content for screening and assessing alcohol use and consequences identified in a meta-analysis conducted by the authors. The resultant 65 items produced were completed by 381 adolescents, and the 33 items brought forward from this exploratory stage were then tested with 827 adolescents.

**Results:** Exploratory analysis indicated that an item based on heavy episodic drinking is most predictive of full AUDIT score > 8 for screening.

The new assessment items have an alpha of 0.92 (adjusted for 10 items), outperforming the best existing instruments.

Confirmatory categorical structural equation modelling supported the exploratory results with the best performing screening item: (In the last 3 months) on how many days did you have six or more drinks on the same occasion?

The assessment items demonstrated excellent model fit: CFI = 0.99, TLI = 0.99, RMSEA = 0.059 (90% CI: 0.056-0.062) with an alpha value of 0.87 (adjusted for 10 items).

**Conclusions:** The significance of this new item bank for screening and assessing alcohol-related consequences in adolescents, recommendations for further validation and applicability to different practice settings will be discussed.

### O15 Measuring the impact of employment stresses on mental health and alcohol consumption in young people

#### Andrew Divers^1*^, Fiona Helyer^2^, Clifford Johnson^1^, Hazel Wright^1^, Dorothy Newbury-Birch^1^

##### ^1^Teesside University, Middlesbrough, UK; ^2^Middlesbrough Council, Middlesbrough, UK

###### **Correspondence:** Andrew Divers (a.divers@tees.ac.uk)

*Addiction Science & Clinical Practice* 2023, **18(Suppl 1)**: O15

**Background:** The COVID-19 pandemic has affected many aspects of our lives, and the impact of this is far from over. This paper will examine both the temporary and lasting changes to factors affecting alcohol consumption—the amounts consumed as well as the patterns, habits, and attitudes around this—in the light of changing pressures as we move through various stages of the pandemic.

**Materials and methods:** Data for this study has been gathered through a combination of purposive, semi-structured interviews and online questionnaires. In total, 106 participants have been interviewed and 1242 responses to online questionnaires have been gathered. Both interview and qualitative survey data subjected to thematic analysis are presented here.

**Results:** The study is still ongoing, but initial findings suggest that a significant contributor to deleterious mental health and wellbeing in young people is their employment experience—from the stress of being unable to find secure employment (and associated financial concerns) to unique demands and concerns over safety brought about by the workplace during the pandemic.

**Conclusions:** One of the primary areas impacting mental (and in some cases physical) health during this time has been the demands and changes to employment, especially for young people. The effect that this has and will continue to have will be explored, and implications for future brief interventions will be discussed.

### O16 Decoupling SBIRT from in person visits: understanding factors influential to engaging college students in electronic health screens & motivational interventions

#### W. Turner*, J. Kamon, S. G. Mitchell, L. B Monico

##### Center for Behavioral Health Integration O5602 United States, Friends Research Institute, Baltimore, MD 21201, USA 

###### **Correspondence:** W. Turner (wincturner@gmail.com)

*Addiction Science & Clinical Practice* 2023, **18(Suppl 1)**: O16

**Background:** High rates of substance use, mental health risk, and low treatment engagement for college-aged young adults (18–24 years old) is a major healthcare problem in the US. While prior screening, brief intervention, and referral to treatment (SBIRT) approaches with this population have been commonly embedded in campus counseling/health centers and targeted screening with those seeking services, COVID-19’s disruption and displacement of college students required alternative methods for reaching college students.

**Materials and methods:** Data presented draw from a CSAT-funded college SBIRT project conducted across 4 college campuses in Vermont, US September 2020 through March 2022. Due to varied states of campus closure during this period, SBIRT was decoupled from in-person clinic visits and a universal screening approach was undertaken using virtual platforms for screening as well as hybrid approaches for treatment delivery.

**Results:** Campus based wellness staff used a range of targeted outreach activities to find and screen naturally occurring sub-communities of students including campus wide email requests, QR codes on billboards, class & dormitory visits and events. Results indicated not just the feasibility and acceptability of using these outreach approaches but a high overall prevalence of MH and SU together (61%) with use of alcohol, cannabis, or other drugs (18%), mental health risk for depression (25%), and anxiety (36%). In addition, of those students screened 12% had thoughts of self-harm.

**Conclusions:** College students hearing about the wellness program through a class, residence hall, sports team or campus activity will initiate and complete the electronic health/wellness screening. Students identified with risks during screening for depression, anxiety, and/or substance use were willing to engage in brief motivational conversations with the wellness staff after receiving one or several of the following: email response with health resources, calendar link and/or incentives for engagement.

### O17 Adolescent behavioral responses to COVID-19 and the development of the Pandemic Response Index

#### Shannon Gwin Mitchell^1*^, Laura B. Monico^1^, Jan Gryczynski^1^, Kaitlyn Garrison^1^, Tyler Ross^1^, Kevin O’Grady^2^

##### ^1^Friends Research Institute, Baltimore, MD 21201, USA;^2^QuantAid

###### **Correspondence:** Shannon Gwin Mitchell (smitchell@friendsresearch.org)

*Addiction Science & Clinical Practice* 2023, **18(Suppl 1)**: O17

**Background:** This analysis seeks to understand the impact of the COVID-19 pandemic-related lockdowns on substance use behaviors among a sample of adolescent primary care patients.

**Materials and methods:** Data for this analysis were compiled from a large RCT testing the effectiveness of a screening, brief intervention, and referral to treatment (SBIRT) package among adolescents (aged 12–17, inclusive) in primary care. Recruitment largely ceased in March 2020 but 249 individuals completed a 12-month follow-up survey that also included a COVID-19 specific questionnaire. These items were analyzed as five distinct indices, referred to as the Pandemic Response index (PRI): Positive Actions, Negative Actions, Anti-Social Behavior, Family Conflict, and Family Stress. Participants also completed the S2BI, ASSIST, and BSTAD for substance use. Four separate logistic regression analyses were conducted on each of the following outcomes: tobacco, alcohol, marijuana, and any substance use.

**Results:** The sample was primarily white (70%), non-Hispanic (63%), with an average age of 14.2 years. With regard to the PRI, the odds of tobacco use decreased 50% with each 1 SD increase in Positive Action score, and increased 82% with each 1 SD increase in Family Stress score. The odds of alcohol use increased 60% with each 1 SD increase in Negative Actions score, and increased 67% with each 1 SD increase in Antisocial Behavior score. The odds of any substance use increased 45% with each 1 SD increase in Family Stress score. There were no significant PRI predictors of marijuana use.

**Conclusions:** Adolescents are a unique and vulnerable population to consider during the COVID-19 pandemic, given the loss of structural support provided by daily activities and connections with other youth and adults outside the home. Greater behavioral and mental health support should be targeted toward adolescents experiencing negative responses in order to decrease the likelihood of substance use.

### O18 Phone based brief intervention for alcohol use reduction during a COVID-19 pandemic: a telenursing care proposal in primary health care in Brazil

#### Divane de Vargas^*^, Caroline Figueira Pereira, Erika Gisseth León Ramírez, Jose'Adelmo da Silva Filho, Sheila Oliveira Ramos

##### ^1^School of Nursing, University of São Paulo, São Paulo, Brazil

###### **Correspondence:** Divane de Vargas (vargas@usp.br)

*Addiction Science & Clinical Practice* 2023, **18(Suppl 1)**: O18

**Background:** Studies from Brazil showed that more than 50% of the population had an increase in alcohol consumption during the first year of the COVID-19 pandemic, which may result in complications arising from harmful use of this substance and increased need for specialized medical care. This can be most challenging for emerging developing countries such as Brazil, where political and socioeconomic conditions, as well as a scarcity of mental health care services, had been experienced even before the COVID-19 pandemic. Therefore, preventive strategies to prevent mental health disorders, as well as alcohol misuse in the population, when face-to-face contact is restricted, should be implemented.

**Objective:** This analyzed the impact of a one-session remote brief intervention, carried out by nurses through a telephone approach to reduce alcohol use in Primary Health Care patients in Brazil.

**Methods:** Quasi-experimental study conducted with 1270 participants who answered the Alcohol Use Disorders Identification Test (AUDIT C). This study considered delivering the phone-based brief intervention to those whose AUDIT scores indicated risky or harmful alcohol use. All participants who received the phone-based intervention were followed 90 and 180 days after the intervention.

**Results:** A positive effect of the phone-based brief intervention in alcohol use reduction was observed in all follow-ups (¬µ = 1.57 p < 0.001).

**Conclusions:** The results suggest that the phone-based brief intervention delivered by primary health care nurses is a potential alternative for preventive care in mental health and alcohol misuse, in situations where the face-to-face screening and brief intervention among primary health care patients were restricted.

### O19 Adapting a primary care collaborative care intervention trial in response to the COVID-19 pandemic: remote recruitment and assessments in the Subthreshold Opioid Use Disorder Prevention (STOP) trial

#### Jennifer McNeely^1*^, Rebecca Stone^2^, Noa Appleton^1^, Amanda M. Bunting^1^, Geetha Subramaniam^3^, Jennifer McCormack^4^, Tobie Kim^4^, Ashley Case^4^, Margaret Kline^4^, Rebecca Price^4^, Eve Jelstrom^4^, Travis Lovejoy^5^, Lillian Gelberg^6^, Jane M. Liebschutz^7^

##### ^1^Department of Population Health, New York University Grossman School of Medicine, New York, NY 10016, USA; ^2^Department of Psychiatry, New York University Grossman School of Medicine, New York, NY 10016, USA; ^3^National Institutes of Health, National Institute on Drug Abuse, Bethesda, MD 20852, USA ; ^4^The Emmes Company, Rockville, MD, USA; ^5^Department of Psychiatry, Oregon Health & Science University, Portland, OR 97239, USA; ^6^Department of Family Medicine, David Geffen School of Medicine at UCLA, Los Angeles, CA 90095, USA; ^7^Division of General Internal Medicine, Center for Research On Health Care, University of Pittsburgh School of Medicine, Pittsburgh, PA 15213, USA

###### **Correspondence:** Jennifer McNeely (jennifer.mcneely@nyulangone.org)

*Addiction Science & Clinical Practice* 2023, **18(Suppl 1)**: O19

**Background:** The Subthreshold Opioid Use Disorder Prevention (STOP) Trial is a 5-site randomized controlled trial of a primary care intervention for risky opioid use. The study tests the effectiveness of a collaborative care intervention consisting of brief advice delivered by the primary care provider (PCP), telephone health coaching (2–6 sessions), and an in-clinic nurse care manager (12 months). Recruitment began in early 2021, and pandemic-related changes at the study sites (telehealth visits, restrictions on research staff in clinic) required adaptation of the original plans for in-person recruitment, enrollment, assessments, and PCP interventions.

**Methods:** This cluster-randomized trial enrolls PCPs and their adult patients (18 +) who have opioid misuse but not moderate-severe opioid use disorder. Key adaptations to study procedures were: (1) remote patient recruitment, using a combination of messages sent through the electronic health record (EHR), mailed letters, email, and text messages inviting patients to take an on-line prescreening assessment; (2) delivery of PCP brief advice during telehealth visits or phone calls within 10 business days of enrollment; and (3) fully remote computerized study procedures for screening and assessments.

**Results:** The study is ongoing, and results are reported for the first 12 months. A total of 101,233 invitations to prescreen were sent to patients identified in the EHR, and 20,148 completed the prescreener, representing a 20% response rate. Of those completing prescreening, 2.3% prescreened eligible, of which 36% were eligible for the study, and 83% enrolled. PCPs frequently delivered brief advice with phone calls that were not part of a medical visit. Completion rates for on-line monthly assessments ranged from 94–99%.

**Conclusions:** Recruitment for this primary care study has been challenged by relying on remote methods, primarily due to low response to invitations to prescreen. For enrolled patients, participation in remote assessments has been high, demonstrating good acceptability of this approach.

### O20 Alcohol use and binge drinking during COVID-19 pandemic: a multicentre cross-sectional study in Brazil

#### Erika Gisseth León Ramírez^1*^, Divane de Vargas^1^, Jaqueline Ribeiro Magalhães^1^

##### ^1^Psychiatric Nursing Department, School of Nursing University of São Paulo, São Paulo, Brazil 

###### **Correspondence:** Erika Gisseth León Ramírez (egleonr@usp.br)

*Addiction Science & Clinical Practice* 2023, **18(Suppl 1)**: O20

**Background:** Binge drinking is a type of harmful use defined as the use of about 60 g of alcohol in about 2 h. The increase in binge drinking intensified with the restriction measures arising of COVID-19 Pandemic. There is evidence that social isolation has potentiated stress conditions, with consequences for the mental health of the population, such as increased the alcohol use. In Brazil, although there are few publications about the binge drinking associated with the isolation period, there is evidence of an increase about 17% in the alcohol use by Brazilians during the pandemic. Therefore, this study aims to investigate the alcohol use, binge drinking and correlated factors in patients from primary health care services in the city of São Paulo-Brazil.

**Methods:** This is a multicentre cross-sectional study carried out between December 2020 and March 2022, conducted with 1285 patients of primary health care services in several regions of the city of São Paulo. The Alcohol use form was applied, with questions about alcohol use before and during the pandemic. The alcohol use classification was performed using the short version of the Alcohol Use Disorders Identification Test (AUDIT-C).

**Results:** A large majority (83.1%) of the respondents reported consuming alcohol in the previous three months, of these 30% (44.9% male and 55.1% female) reported engaging in binge drinking at least once in the previous three months. Using logistic regression analyses, results showed that having depression, occupational levels, and increase of alcohol during the pandemic, were all significantly and independently associated with binge drinking.

**Conclusions:** Our findings indicate that, situational, social and psychological factors are important determinants of excessive alcohol consumption, and it could be used as a potential target for interventions to reduce alcohol use and prevent complications arising from this consumption.

### O21 Early identification and brief intervention for alcohol use disorders: the training programme on EIBI of the Local Health Unit of Salerno, Italy

#### Claudia Gandin^1*^, Aniello Baselice^2^, Silvia Ghirini^1^, Alice Matone^1^, Antonio De Luna^2^, Emanuele Scafato^1^

##### ^1^National Observatory on Alcohol, National Centre on Addictions and Doping, Istituto Superiore di Sanità, Rome, 00161, Italy; ^2^Department of Addictions, Local Health Unit Salerno, Campania Region, Italy

###### **Correspondence:** Claudia Gandin (claudia.gandin@iss.it)

*Addiction Science & Clinical Practice* 2023, **18(Suppl 1)**: O21

**Background:** "Rete IPIB-ASL Salerno" is a 4 year programme on EIBI (Identificazione Precoce e Intervento Breve ‚Äì IPIB in Italian) for Alcohol Use Disorders (AUDs) in Primary Health Care (PHC) promoted by the Addiction Department of the Salerno Local Health Unit (LHU), Campania Region and implemented in partnership with the Istituto Superiore di Sanità.

– To develop and implement a local training programme on IPIB for AUDs for PHC professionals;

– To create a network of professionals of the Salerno LHU qualified on IPIB for AUDs and other lifestyles behaviours.

**Materials and methods:** Steps of activities:

1. Analysis of the resources on alcohol prevention-interventions for activation of training (mapping the Salerno LHU services);

2. Implementation of the training on IPIB for AUDs and other lifestyles behaviours of health professionals starting from the PHEPA project standard (Primary Health Care Project on Alcohol) plus additional units (such as Unit 2 ‚ Äú Attitudes to alcohol‚ Äù) from the new World Health Organization alcohol brief intervention training manual for primary care (2017).

3. Creation of a local network of professionals skilled on IPIB model.

**Results:** Overall, training reached about 500 professionals of the Salerno LHU (who have completed the training). The attitudes on alcohol of professionals before/after the training and the merged needs will be summarized (the lack of sufficient training, of e-interventions during the COVID-19 emergency, of role legitimacy and of time as main obstacles for the IPIB implementation; the availability of a multidisciplinary team, the network and its coordination, the guidelines-protocols-tools as facilitators).

**Conclusions:** For self-replicating / self-maintaining the training activity over time and for creating a consolidated network of trainers qualified on IPIB for AUDs and other lifestyles behaviours it is essential:

– To ensure training skills and knowledge on IPIB for AUDs for all professionals working in PHC settings;

– To support the coordination of the network at local level involving different settings for different target populations.

### O22 Effects of adolescent SBIRT education using simulated learning technology in health professional training

#### Jackie Sheridan-Johnson^1*^, Weiwei Liu^1^, Hildie Cohen^2^, Tracy L. McPherson^1^

##### ^1^Public Health, NORC at the University of Chicago, Chicago, IL 60603, USA; ^2^Health Sciences, NORC at the University of Chicago, Chicago, IL 60603, USA

###### **Correspondence:** Jackie Sheridan-Johnson (sheridan-jackie@norc.org)

*Addiction Science & Clinical Practice* 2023, **18(Suppl 1)**: O22

**Background:** Social workers, nurses, and other health professionals can help prevent and reduce substance use among youth by using the screening, brief intervention, and referral to treatment (SBIRT) model in care settings. NORC at the University of Chicago, along with leading professional education experts, developed and tested an adolescent SBIRT curriculum for use in nursing, social work, and interprofessional education. Since 2015, the curriculum has been implemented with more than 15,000 students in over 600 U.S. programs. This study evaluated the impact of the education on students' attitudes towards working with people who drink alcohol; perceived readiness, confidence, competence; knowledge, and skills.

**Materials and methods:** Students completed a pre-training survey, received adolescent SBIRT education including an online simulation, and a post-training survey. A pretest–posttest design was used to investigate the effects of the education on student attitudes, confidence, competence, readiness, knowledge, and skills. The sample included 33 schools with 1646 students. Paired t-tests were conducted to evaluate overall differences between pre- and post- measures. Subgroup (e.g., undergraduate/graduate, prior training) differences in outcomes were evaluated using independent sample t-tests and OLS regressions. Separate OLS regression models were conducted for undergraduate and graduate students.

**Results:** All outcome measures were significantly higher after SBIRT education. Undergraduate students showed significantly higher improvement than graduate students in confidence, competence, and readiness. Controlling for other demographic covariates, graduate students without prior SBIRT training showed significantly higher improvement in competence, confidence, readiness, and knowledge compared to those with prior training. No such differences were shown for undergraduate students.

**Conclusions:** Findings suggest that adolescent SBIRT education including simulation-based training can positively build students knowledge and skills and boost their confidence in implementing adolescent SBIRT. Results also suggest the importance of tailoring training to suit the needs of different subgroups with different past training and experiences.

### O23 Validity of the Single-Item Screen-Cannabis (SIS-C) for cannabis use disorder screening in routine care

#### Theresa E. Matson*, Gwen T. Lapham, Jennifer F. Bobb, Malia Oliver, Kevin A. Hallgren, Emily C. Williams, Katharine A. Bradley

##### Kaiser Permanente Washington Health Research Institute, Seattle WA 98101, USA

###### **Correspondence:** Theresa E. Matson (tessa.e.matson@kp.org)

*Addiction Science & Clinical Practice* 2023, **18(Suppl 1)**: O23

**Background:** Cannabis use is prevalent and increasing. Frequent cannabis use intensifies risk of cannabis use disorder (CUD). CUD is underrecognized in medical settings, but a validated single-item cannabis screen could increase recognition and care. The study aimed to validate the Single-Item Screen-Cannabis (SIS-C), administered and documented in routine primary care, compared to a confidential reference standard of CUD.

**Methods:** This validation study was conducted in an integrated health system in Washington, where adult cannabis use is legal. Participants were adult patients screened for cannabis use in primary care who also responded to a confidential survey (N = 1688; 34% response rate). The SIS-C asks about frequency of past-year cannabis use with responses (none, less than monthly, monthly, weekly, daily/almost daily) documented in patients medical records. The SIS-C was compared to patient response on the Diagnostic and Statistical Manual, 5th edition (DSM-5) Composite International Diagnostic Interview (CIDI) for past-year CUD (i.e., reference standard), completed on the survey. Analyses estimated screening performance (e.g., sensitivity, specificity, area under receiver operating characteristic curves [AUC]) of the SIS-C. Analyses were weighted, accounting for survey design and nonresponse, to obtain estimates representative of the health system primary care population.

**Results:** 6.6% of patients met •2 criteria for past-year CUD, including 1.9% who met •4 criteria for moderate-severe CUD. The SIS-C demonstrated strong validity for identifying any CUD (AUC 0.89 [95% CI: 0.78–0.96]) and moderate-severe CUD (AUC 0.95 [0.94–0.96]). A threshold of less than monthly cannabis use had the best balance of sensitivity (0.88) and specificity (0.83) for detecting any CUD; a threshold of monthly use had the best balance of sensitivity (0.96) and specificity (0.89) for detecting moderate-severe CUD.

**Conclusion:** The SIS-C had excellent performance when used in routine care as a screen to identify patients at risk of CUD for brief intervention or further assessment.

### O24 Usability of a brief intervention combined with a gamified webapp to improve retention to the addiction treatment in patients with alcohol-related liver disease

#### Elsa Caballeria Lamora^1*^, Clara Oliveras^1^, Maria Teresa Pons-Cabrera^1^, Neus Freixa^1^, Pol Bruguera^1^, Laura Nuño^1^, Anna Lligoña^1^, Alexandra Estruch^2^, Oscar García-Pañella^2,3^, Soraya Sabate^1^, Ana Isabel López-Lazcano^1^, Cristal Martínez^1^, Antoni Gual^1^, Mercè Balcells-Oliveró^1^, Hugo López-Pelayo^1^

##### ^1^GRAC, Addictions Unit, Department of Psychiatry, Clinical Institute of Neuroscience, Hospital Clínic, RETICS, University of Barcelona, Institut d'Investigacions Biomèdiques August Pi i Sunyer (IDIBAPS), Barcelona, Spain; ^2^Cookie Box S.L, Barcelona, Spain; ^3^Escuela de Nuevas Tecnologías Interactivas, Barcelona, Spain

###### **Correspondence:** Elsa Caballeria Lamora (caballeria@recerca.clinic.cat)

*Addiction Science & Clinical Practice* 2023, **18(Suppl 1)**: O24

**Background:** Retention to the addiction treatment in patients with alcohol-related liver disease (ARLD) is scarce despite the crucial role of abstinence. With the aim of improving treatment retention in these patients, and using a co-creational methodology, we designed an intervention consisting in a gamified Webapp (6 weeks of duration) and a brief 2-sessions face-to-face motivational intervention. We present the results regarding the usability and satisfaction of the patients with the intervention.

**Materials and methods:** Two co-creation sessions were organized, with the participation of 40 people (professionals, patients and patients relatives) to collect information to design the intervention format and contents. Once the first version of the intervention (brief face-to-face intervention + Webapp) was designed, a usability study was conducted. For that, 10 patients with ARLD and 10 professionals were recruited. They received a weekly call to answer an open interview regarding the completed module of the webapp, and completed two questionnaires regarding usability (System Usability Scale, SUS; Post-Study System Usability Questionnaire, PSSUQ).

**Results:** Receiving personalized feedback, the adaptation of the intervention storytelling to the progress of the patient, providing additional content and sources, adding interactive elements to the intervention and using a broad variety of exercises are the most valued aspects of the intervention. The brief intervention was considered a good first step before being referred to the Addictions Unit, with the information provided being of quality and useful. Regarding the Webapp, participants reported that it was easy to use, with a pleasant interface, with clear information and the expected capabilities and contents.

**Conclusions:** Including the targeted patients of the intervention and professionals to the design process was feasible. Complementing the brief intervention with a gamified Webapp is promising, although the efficacy still needs to be studied (we expect to have the first results on efficacy by august).

### O25 Understanding what strategies Dry January participants use to temporarily abstain from alcohol

#### Anna L Butters^1*^, Matt Field^1^, Inge Kersbergen^2^, John Holmes^2^

##### ^1^Department of Psychology, University of Sheffield, Sheffield, South Yorkshire, S1 2LT, UK; ^2^School of Health and Related Research, University of Sheffield, Sheffield, S1 4DA, UK

###### **Correspondence:** Anna L Butters (abutters1@sheffield.ac.uk)

*Addiction Science & Clinical Practice* 2023, **18(Suppl 1)**: O25.

**Background:** Remaining fully abstinent during temporary abstinence challenges such as Dry January is associated with greater ongoing reductions in alcohol consumption. Participants in these challenges use various strategies to avoid drinking alcohol, including goal setting, self-monitoring, public commitment, and restructuring their social environments. It is unclear how use of these strategies affects abstinence during Dry January. This exploratory research aimed to identify which strategies Dry January participants use to maintain abstinence and evaluate to what extent strategy use is associated with abstinence (or alcohol consumption) during the month.

**Materials and methods:** We first identified 14 strategies to avoid drinking in collaboration with past Dry January participants. Then, 105 people who had registered for Dry January completed baseline and post-January questionnaires. We measured alcohol consumption (AUDIT-C), motivation to change, drink-refusal self-efficacy (DRSE) and how frequently participants used the 14 strategies to avoid drinking. Data were analysed using linear, logistic and poisson regression models.

**Results:** There was considerable variation in the number and frequency of strategies used during Dry January. Two strategies, Public commitment and Restructuring the social environment were independently associated with greater reduction in AUDIT-C over the course of January. No strategy was significantly associated with total abstinence. The number of strategies participants used was not significantly associated with total abstinence or change in AUDIT-C.

**Conclusions:** Making a public commitment to temporary abstinence or restructuring one social environment may help to limit any consumption that occurs when someone is attempting to abstain during Dry January. Recommending the use of these strategies during Dry January may improve drinking outcomes among participants. Considerable variation in the use of strategies between participants indicates that further research is needed to understand how and why people use certain strategies.

### O26 Investigating for whom brief substance use interventions are most effective: an individual participant data meta-analysis

#### Maria L. Schweer Collins^1*^, Nicholas J. Parr^2^, Rich Saitz^3,4^, Emily E. Tanner-Smith^1,5^

##### ^1^University of Oregon, Prevention Science Institute, Eugene, OR, USA; ^2^U.S. Department of Veterans Affairs Evidence Synthesis Program Coordinating Center, VA Portland Health Care System, Portland, OR, USA; ^3^Department of Community Health Sciences, Boston University School of Public Health, Boston, MA, USA; ^4^Clinical Addiction Research and Education Unit, Section of General Internal Medicine, Boston University School of Medicine; Grayken Center for Addiction, Boston Medical Center, Boston, MA, USA; ^5^University of Oregon, Counseling Psychology and Human Services Department, University of Oregon, Eugene, OR, USA

###### **Correspondence:** Maria L. Schweer Collins (mschweer@uoregon.edu)

*Addiction Science & Clinical Practice* 2023, **18(Suppl 1)**: O26

**Background:** Brief interventions (BIs) are one evidence-based and widely implemented prevention strategy to address substance use. A large body of literature has examined the efficacy of BIs for addressing alcohol and other drug use and shows that BI effects can vary widely across studies, including minimal or inconsistent efficacy among adolescent and aging adults, persons experiencing unstable housing, women, and those with minoritized racial and ethnic identities. Due to these critical gaps, the objective of this individual participant data (IPD) meta-analysis was to explore the types of patients for whom BIs delivered in general healthcare settings are more or less effective on a range of outcomes.

**Methods:** In this IPD meta-analysis, we synthesized evidence from 29 randomized trials, drawn from a larger aggregate data meta-analysis. The meta-analysis was pre-registered with PROSPERO (#CRD42018086832). Analyses were carried out using a two-stage IPD meta-analysis approach.

**Results:** Trials were conducted in the United States and Canada (k = 17; 55%) and internationally (k = 13, 45%). For alcohol consumption at 3-months post-treatment, BIs led to significant reductions in binge alcohol consumption (0.09, 95% CI [0.03, 0.14]) and frequency of alcohol consumption (0.10, 95% CI [0.03, 0.17]) among females. BIs also led to a significant reduction in frequency of alcohol consumption (0.16, 95% CI [0.09, 0.22]) among patients with educational attainment below high school level. At 6-months post-treatment, patients with non-White identities showed BI effects on significant reductions in cannabis consumption quantity relative to control (0.07, 95% CI [0.05, 0.10]). Complete results will be presented.

**Conclusions:** When delivered in general healthcare settings, females and individuals with below high school educations show modest, beneficial reductions in alcohol consumption. There is limited evidence of benefits of BI on drug use outcomes, although non-White individuals show BI-driven reductions in cannabis use following intervention.

### O27 Barriers to seeking treatment for alcohol use disorders: the role of severity of alcohol use and gender

#### Sara Wallhed Finn*, Anna Mejldal

##### University of Southern Denmark, Odense, Denmark & Karolinska Institutet, Stockholm, Sweden

###### **Correspondence:** Sara Wallhed Finn (sara.wallhed-finn@ki.se)

*Addiction Science & Clinical Practice* 2023, **18(Suppl 1)**: O27

**Background:** A minority of all individuals with alcohol use disorders (AUD) seek treatment. Since the suffering from AUD has severe consequences for both the individual and for society, knowledge about barriers for treatment seeking is of importance. Most studies of barriers this far have been conducted in the USA or the UK. There is a need for studies from other contexts.

**Aim:** To investigate barriers to treatment seeking for AUD.

The specific aims are to investigate:

1. Barriers to AUD treatment at different levels of alcohol use.

2. Gender differences regarding barriers to AUD treatment.

**Methods:** Study design: Cross-sectional. Participants: 1594 representative Danish adults from the general population aged 30 ‚Äì 65 years. An online questionnaire was administrated by a market research company. The questionnaire covered demographic data, barriers to treatment and level of alcohol use. Analyses were performed by means of chi-2 test and logistic regression.

**Results:** The most common barriers were related to stigma. Those with higher severity of alcohol use are more like to endorse a wish to handle alcohol problems themselves and to report treatment related barriers. Women with high severity of alcohol use, endorsed higher fear of the consequences than men.

**Conclusion:** Individuals with low alcohol use report a willingness to seek professional treatment if a problem occur; however, it seems that this decrease if severe alcohol use is present, in particular among women. Especially among individuals with high severity of alcohol use there is a need to address gender specific barriers.

### O28 Who benefits from brief motivational intervention among alcohol-intoxicated young adults admitted to the emergency department: a moderation profiles analysis

#### Jacques Gaume^1*^, Molly Magill^2^, Jim McCambridge^3^, Nicolas Bertholet^1^, Olivier Hugli^4^, Jean-Bernard Daeppen Bertholet^1^

##### ^1^Department of Psychiatry-Addiction Medicine, Lausanne University Hospital, 1011 Lausanne, Switzerland; ^2^Center for Alcohol and Addiction Studies, Brown University School of Public Health, Providence, RI 02,912, USA; ^3^Department of Health Sciences, University of York, York, YO10 5DD, UK; ^4^Emergency Department, Lausanne University Hospital, Lausanne, 1011, Switzerland

###### **Correspondence:** Jacques Gaume (jacques.gaume@chuv.ch)

*Addiction Science & Clinical Practice* 2023, **18(Suppl 1)**: O28

**Background:** Little is known about which patients benefit from brief Motivational Interviewing (bMI) for heavy drinking among young adults in the Emergency Department (ED).

**Materials and methods:** We conducted moderation analyses of data from a randomized controlled trial (RCT). Young adults (18–35 years) admitted in the ED with alcohol intoxication (N = 344) were randomized to receive either bMI or brief advice (BA). Outcome was the number of heavy drinking days at short- (1-month) and long-term (12-month) follow-up. We used latent profile analyses to derive participants profiles based on baseline characteristics (gender, age, AUD severity, attribution of ED admission to alcohol use, confidence/importance to change, cognitive discrepancy, positive/negative alcohol expectancies, anxiety, depression, and trait reactance). We then computed negative binomial regressions with an interaction between intervention and dummy-coded profiles.

**Results:** Fit statistics indicated a 4-profile solution. At short-term follow-up, there were significant interactions when comparing Profiles 1 and 2, and Profiles 1 and 3. At long-term follow-up, there was a significant interaction when comparing Profiles 1 and 2. Profile 2 was characterized by high severity, negative expectancies, importance, and discrepancy, low confidence, and low anxiety; those participants benefitted more from bMI at 1-month and 12-month as compared to Profile 1. Profile 3 was characterized as older participants, having the highest severity, negative expectancies, importance, discrepancy, reactance, anxiety and depression, but the lowest confidence; those benefitted more from bMI at 1-month as compared to Profile 1. Profile 1 was characterized as younger participants, having the lowest severity, expectancies, importance, discrepancy, reactance, anxiety and depression, and the highest confidence; those benefitted more from BA.

**Conclusions:** Findings showed important moderation effects within a RCT having shown the effectiveness of bMI on heavy drinking. This suggest that patients characteristics profiles should be considered when implementing bMI.

### O29 Effectiveness of alcohol brief intervention on drinking and blood pressure outcomes in adult patients with hypertension: results from a systematic alcohol screening and brief intervention initiative in a U.S. health system

#### Stacy Sterling^1*^, Felicia W. Chi^1^, Sujaya Parthasarathy^1^, Vanessa A. Palzes^1^, Andrea H. Kline-Simon^1^, Constance Weisner^1^, Derek D. Satre^2^, Yun Lu^1^, Verena E. Metz^1^

##### ^1^Division of Research, Kaiser Permanente Northern California, Oakland, CA, United States, 94612; ^2^Department of Psychiatry and Behavioral Sciences, University of California, 675 18th Street, San Francisco, CA 94107, USA

###### **Correspondence:** Stacy Sterling (stacy.a.sterling@kp.org)

*Addiction Science & Clinical Practice* 2023, **18(Suppl 1)**: O29

**Background:** Many patients with hypertension drink above recommended limits, which may affect disease management and blood pressure (BP) outcomes. Alcohol brief intervention (ABI) in adult primary care has been found efficacious in reducing hazardous drinking. However, effectiveness research in the context of real-world implementation is rare, and little is known about effects of ABI on health outcomes. This study aims to evaluate the effectiveness of ABI in adult primary care on 2- and 5-year drinking and BP outcomes among adults with hypertension, by using advanced causal inference techniques to emulate a target trial.

**Materials and methods:** This population-based observational study was conducted at Kaiser Permanente Northern California, an integrated healthcare system that implemented system-wide alcohol SBIRT in adult primary care in mid-2013. Using longitudinal electronic health record (EHR) data on 72,979 hypertensive patients aged 18–85 who screened positive for unhealthy alcohol use between 2014 and 2017, dynamic marginal structural models with inverse probability weighting for informative censoring were fit to estimate adjusted effects of ABI.

**Results:** Compared with never receiving ABI, receiving ABI whenever screened positive was associated with a greater decrease in heavy drinking days at 2 years (mean difference [95% confidence intervals] = -– 0.21 [– 0.39, – 0.04]); the effect became smaller at 5 years. Receiving ABI whenever screened positive was also associated with better BP outcomes at 2 years, resulting in 6–8% higher odds of having clinically meaningful reduction in BP (i.e., ‚â•3 mmHg reduction from baseline). However, we found no significant ABI effects on BP at 5 years.

**Conclusions:** Our analysis using causal inference techniques suggests that ABI holds promise for reducing drinking and helping to improve health outcomes among adults with hypertension who screen positive for unhealthy drinking. However, more research is needed to understand effect heterogeneity across diverse sub-populations and to study ABI’s long-term public health impact.

### O30 Substance use screening rates and screening results among adult primary care patients with mental health conditions and substance use-related medical conditions

#### Jennifer McNeely^1*^, Aimee Wahle^2^, Margaret Kline^2^, Sarah Wakeman^3^, Timothy Wilens^4^, Dr. Joseph Kannry^5^, Richard N. Rosenthal^6^, Angeline Adam^7^, Noa Appleton^1^, Sarah Farkas^8^, Carmen Rosa^9^, Seth Pitts^2^, Keith Goldfeld^1^, John Rotrosen^8^, Leah Hamilton^10^

##### ^1^Department of Population Health, New York University Grossman School of Medicine, New York, NY 10016, USA; ^2^The Emmes Company, Rockville, MD 20850, USA; ^3^Department of Medicine, Harvard Medical School, Massachusetts General Hospital, Boston, MA 02114, USA; ^4^Department of Psychiatry, Massachusetts General Hospital, Boston, MA 02114, USA; ^5^Division of General Internal Medicine, Icahn School of Medicine at Mount Sinai, New York, NY 10029, USA; ^6^Renaissance School of Medicine, Stony Brook University, Stony Brook, NY 11794, USA; ^7^Department of Psychiatry, University Hospital Lausanne, Lausanne, Switzerland; ^8^Department of Psychiatry, New York University Grossman School of Medicine, New York, NY 10016, USA; ^9^National Institutes of Health, National Institute on Drug Abuse, Bethesda, MD  20852, USA; ^10^Kaiser Permanente Washington Health Research Institute, Seattle, WA 98101, USA

###### **Correspondence:** Jennifer McNeely (jennifer.mcneely@nyulangone.org)

*Addiction Science & Clinical Practice* 2023, **18(Suppl 1)**: O30

**Background:** Patients with mental health conditions (MHCs) and substance-related medical conditions (SRMCs) are at elevated risk for poor health outcomes related to alcohol and drug use. We analyzed substance use screening for patients with these conditions in 6 U.S. primary care clinics that participated in a screening implementation study. Clinics initiated universal screening for adult patients in 2017–2018.

**Methods:** Data were extracted from electronic health records for one year pre- and post-screening implementation, and adults having visits in both years were included. ICD-10 diagnosis codes from the Problem List prior to screening implementation identified MHCs and SRMCs. Screening rates and screen-positive rates were compared for patients with and without MHCs and SRMCs using multivariate logistic regression models adjusted for key demographic characteristics.

**Results:** Of the 39,148 patients meeting inclusion criteria, 29% had MHCs and 58% had SRMCs. Screening for alcohol/drugs was completed by 47% of patients with MHCs and 57% of patients with SRMCs. Patients with MHCs had lower screening rates for alcohol (AOR = 0.43, 95% CI:0.41–0.45) and drugs (AOR = 0.43, 95% CI:0.41–0.45) in comparison to patients without MHCs. Similarly, patients with SRMCs had lower screening rates for alcohol (AOR = 0.41, 95% CI = 0.38–0.43) and drugs (AOR = 0.41, 95% CI = 0.39–0.43). Patients with MHCs were more likely to screen positive for alcohol (AOR = 1.48, 95% CI = 1.37–1.60) and drugs (AOR = 1.55, 95% CI = 1.32–1.83), while patients with SRMCs were more likely to screen positive for alcohol (AOR = 1.39, 95% CI = 1.29–1.50) but not drugs (AOR = 1.08, 95% CI = 0.93–1.27).

**Conclusions:** With a universal screening approach, patients with MHCs and SRMCs were less likely to be screened, and more likely to screen positive for alcohol use, in comparison to patients without these conditions. While there may be unique barriers to screening patients with mental health and substance-related medical conditions, clinics should consider prioritizing them for screening given the substantial burden of unhealthy substance use.

### O31 Natural course of behavioural health risk factors in general hospital patients with at-risk alcohol use over two years

#### Jennis Freyer Adam^1*^, Anika Tiede^1,2^, Sophie Baumann^3^, Filipa Krolo^1,2^, Beate Gaertner^4^, Ulrich John^2,5^

##### ^1^Institute for Medical Psychology, University Medicine Greifswald, Greifswald, Germany; ^2^German Centre for Cardiovascular Research, Site Greifswald, Greifswald, Germany; ^3^Department of Methods in Community Medicine, Institute of Community Medicine, University Medicine Greifswald, Germany; ^4^Robert Koch Institute Berlin, Department of Epidemiology and Health Monitoring, Berlin, Germany; ^5^Department of Prevention Research and Social Medicine, Institute of Community Medicine, University Medicine Greifswald, Germany

###### **Correspondence:** Jennis Freyer Adam (jennis.freyer-adam@med.uni-greifswald.de)

*Addiction Science & Clinical Practice* 2023, **18(Suppl 1)**: O31

**Background:** About 90% of persons who drink alcohol at-risk also report tobacco smoking, physical inactivity and/ or overweight. Co-occurrence of such behavioral health risk factors (BHRFs) more than doubles the risk for chronic disease and mortality. Little is known about the development of BHRFs over time, particularly among general hospital patients with at-risk alcohol use hospitalized for somatic disease or injury. Hospitalization is considered to be a learnable moment for patients. The aim was to investigate whether general hospital patients identified with at-risk alcohol use change their BHRFs during the first two years after hospitalization.

**Materials and methods:** Data from a randomized controlled trial which was approved by the local ethic commission were used (ClinicalTrials.gov: NCT01291693). Eighteen to 64-year-old general hospital patients with at-risk alcohol use were identified through systematic screening (91% participation). Patients with particularly severe alcohol problems were excluded. Of those eligible, 81% provided informed written consent. For this investigation, data of the treatment as usual control group were analyzed (n = 220). Alcohol use measured by the AUDIT-C, tobacco smoking, vegetable and fruit intake, physical activity and body-mass-index were assessed at baseline, after 6, 12, 18, and 24 months. Latent growth models were calculated.

**Results:** Twenty-four months after hospital discharge, participants reported less physical activity (p = 0.04), a higher body-mass-index (p = 0.01), no change in vegetable and fruit intake (p = 0.11), fewer cigarettes per week (p < 0.001), and less alcohol use (p < 0.001) compared to baseline.

**Discussion:** Although alcohol use and tobacco smoking were reduced, energy-balance related BHRFs developed unfavorably or did not improve over 2 years after hospitalization.

**Conclusions:** These findings underline the need of the implementation of multi-behavioral interventions for at-risk alcohol users in routine care.

### O32 Disparities in receipt of alcohol brief intervention: the intersectionality of sex, age and race/ethnicity

#### Sujaya Parthasarathy^1^*, Andrea H. Kline-Simon^1^

##### Division of Research, Kaiser Permanente Northern California, Oakland, CA 94612, USA

###### **Correspondence:** Sujaya Parthasarathy (sujaya.parthasarathy@kp.org)

*Addiction Science & Clinical Practice* 2023, **18(Suppl 1)**: O32

**Background:** Brief intervention (BI) to address unhealthy alcohol use early shows promise but disparities exists across sex, age and race/ethnicity. The Alcohol as a Vital Sign program (AVS) was introduced into the adult primary care workflow to screen patients for unhealthy alcohol use and provide BI as needed. This study examines BI rates by sex, age, and race/ethnicity, and their intersectionality, incorporating important patient characteristics.

**Materials and methods:** This population-based observational study included adult patients (N = 287,551) screening positive for unhealthy alcohol use between 2014 and 2017 during routine alcohol screening in internal medicine, family practice, and urgent care clinics in an integrated health delivery system. Measures included unhealthy alcohol use and receipt of BI, and patient and provider demographics which were obtained from electronic health records.

**Results:** Multilevel logistic regression was used to examine the likelihood of receiving BI accounting for clustering of patients within providers. Women had lower odds of receiving BI than men in all age groups. Women also had lower odds of receiving BI than men in all racial/ethnic groups; the sex differences in odds of receiving BI were greater for the Latino/Hispanic group (OR [95% CI] for women vs. men = 0.69 [0.66, 0.72]) and smaller for the Asian/Pacific Islander group (OR [95% CI] for women vs. men = 0.76 [0.72, 0.81]).

**Conclusions:** Development of primary care-based alcohol BI approaches that address sex disparities are critical in order to ensure equitable access to this important preventive service.

### O33 The efficacy of iCBT added to treatment as usual for alcohol dependent patients in primary care: a randomized controlled trial

#### Karin Hyland^1*^, Anders Hammarberg^1,3^, Erik Hedman-Lagerlöf^1,4^, Magnus Johansson^2,3^, Philip Lindner^1,3^, Sven Andreasson^2,3^

##### ^1^Department of Clinical Neuroscience, Karolinska Institutet, 17177 Stockholm, Sweden; ^2^Department of Public Health Sciences, Karolinska Institutet, 17177 Stockholm, Sweden; ^3^Centre for Dependency Disorders, Stockholm Health Care Services, Stockholm County Council, Stockholm, Sweden; ^4^Gustavsberg Primary Health Care Center, Stockholm, Sweden

###### **Correspondence:** Karin Hyland (karin.hyland@regionstockholm.se)

*Addiction Science & Clinical Practice* 2023, **18(Suppl 1)**: O33

**Background:** Most alcohol dependent persons have a moderate level of dependence. Treatment seeking in this group is low, mainly due to stigma and because treatment in specialized care is seen as unappealing. This group is more positive to seeking treatment in primary care. General practitioners (GP) hesitate to engage in this area due to time constraints and uncertainty regarding their competence. To lessen the morbidity associated with alcohol dependence and encourage GPs to raise questions about alcohol, they need to have access to treatment they find applicable and feasible to use.

**Materials and methods:** A two group, parallel, randomized controlled superiority trial with a 1:1 allocation. 264 individuals fulfilling ICD-10 criteria for alcohol dependence were randomized to an Internet-based Cognitive Behavioral Treatment program (iCBT) added to treatment as usual (TAU) or TAU only. GPs at 14 primary care centers were offered a 1-h training in giving feedback on assessments and biomarkers and pharmacotherapy prior to the study. Primary outcomes were change in weekly alcohol consumption and heavy drinking days at 3- and 12 months follow-up compared with baseline, as measured with timeline follow back. Secondary outcomes were severity of dependence, consequences of drinking, psychological health and biomarkers.

**Results:** Intention-to-treat analysis (n = 132 + 132) failed to demonstrate improved outcomes when iCBT was added to TAU. The per protocol analysis (n = 102 + 132) however finds that, when the combination actually occurs, iCBT + TAU led to reduced alcohol consumption.

**Conclusions:** The participants had considerable problems with alcohol and health and a majority reduced their alcohol use considerably as well as symptoms of dependence, anxiety and depression at follow-ups. Access to a treatment method that does not take time or require expertise might increase the likelihood that questions about alcohol are asked and contribute to the development of a treatment system where primary care is the base of treatment.

### O34 Brief interventions in decreasing alcohol use in users of a Family Health Unit

#### Tereza Maria Mendes Diniz de Andrade Barroso^1*^, Fernanda Matos Fernandes Castelo Branco^2^, Ferreira Ana Cristina Ferreira^3^

##### ^1^Mental Health Departement, Nursing School Coimbra/Health Sciences Research Unit: Nursing (UICISA: E), Coimbra, Portugal; ^2^University of Federal do Amapá, Oiapoque, AP, Brail; ^3^Family Health Unit Rainha Santa Isabel, Coimbra, Portugal

###### **Correspondence:** Tereza Maria Mendes Diniz de Andrade Barroso (tesouraria@esenfc.pt)

*Addiction Science & Clinical Practice* 2023, **18(Suppl 1)**: O34

**Introduction:** In the Portuguese health system, Primary Health Care (PHC) is the first level of access of individuals, families, and communities to the health system. PHC professionals occupy a privileged position and play a key role in the identification and screening of individuals with hazardous and harmful alcohol consumption, and/or likely alcohol dependence for adequate evaluation, diagnosis, and treatment interventions. Despite this, BIs are not widely used in clinical practice.

**Objective:** To evaluate the effect of brief interventions in decreasing alcohol use in users of a Family Health Unit in the urban region of Portugal.

**Methodology:** Pre-experimental study, before and after evaluation (four months) of 205 users (single group). The SBIRT protocol was implemented and was used the Alcohol Use Disorders Identification Test (AUDIT).

**Results:** In the first assessment, 189 (92.2%) were in zone I; 15 (7.3%) in zone II and 1 (0.5%) in zone IV, this was excluded and referred to the family doctor, with diagnosis and referral for specialized unitIn. In the follow-up, four months after the interventions with the 15 users who scored zone 2, there was a sample loss of 5, showing that 6 (60%) scored zone I, 3 (30%) zone II and 1 (10%) zone III. The results of the Wilcoxon test (before and after the BIs) show that, the risk level after the intervention is lower than before the intervention (Z = -1,402; p = 0,161).

**Conclusion:** The results show a positive effect in reducing the alcohol consumption level of risk associated with the intervention performed. Future studies should be conducted with a bigger sample, with a control group and increasing the time between the implementation and the evaluation. Brief interventions are effective resources in the early detection of alcohol use, which are necessary for dissemination in primary health care.

### O35 Managing alcohol problems through GP practices: referrals to and engagement with a specialist alcohol service

#### Andrea Mohan^1*^, Clare Sharp^2^, Danielle Mitchell^2^, Niamh Fitzgerald^2^

##### ^1^School of Health Sciences, University of Dundee, Dundee, UK; ^2^Institute of Social Marketing and Health, University of Stirling

###### **Correspondence:** Andrea Mohan (amohan001@dundee.ac.uk)

*Addiction Science & Clinical Practice* 2023, **18(Suppl 1)**: O35.

**Background:** General Practitioners (GPs) see a large proportion of the general population and are ideally placed to identify people with alcohol problems. Many GPs will ask patients about their alcohol use if there is a suspected alcohol problem, and provide brief interventions or refer patients to specialist alcohol treatment services. For various reasons, there remains groups of people who do not engage with specialist alcohol services; it is important to understand how to improve this engagement. We conducted a qualitative study that focused on a specialist alcohol service, the Primary Care Alcohol Nurse Outreach Service (PCANOS), that was closely linked to GP practices in Glasgow, Scotland, and supported patients who had low engagement with other alcohol services.

**Materials and methods:** From September 2020 to June 2021, we conducted 25 semi-structured interviews with staff and patients from six GP practices in Glasgow, to explore their views and experiences of managing or receiving support for alcohol problems in primary care. Interviews were transcribed and data were analysed thematically. We present findings relating to the process of referring patients to PCANOS and engaging patients with the service.

**Results:** Most referrals to PCANOS were made by GPs after speaking to the patients about their alcohol use. Speedy referrals were facilitated by the close working relationship practices had with PCANOS. Addiction Nurses (ANs) employed by PCANOS phoned patients as soon as referrals were received and visited patients in their homes to provide specialist care. The ANs used a non-judgemental, person-centred approach and built therapeutic relationships to engage patients with the service.

**Conclusions:** Speedy patients referrals and a person-centred approach to care were essential to patients engaging with PCANOS. It may be beneficial for more GP practices to work collaboratively with specialist alcohol services to appropriately support patients in need to more intensive treatment.

### O36 Primary care provider and U.S. Veteran perspectives on barriers and facilitators to alcohol-related care and ideas for improvement in the Veterans Health Administration

#### Rachel L. Bachrach^1*^, Matthew Chinman^1^, Nicole M. Beyer^2^, Angela Phares^1^, Keri L. Rodriguez^2^, Kevin L. Kraemer^3^, Emily C. Williams^4,5^

##### ^1^Center for Health Equity Research and Promotion; Mental Illness Research, Education, and Clinical Center; VA Pittsburgh Healthcare System, Pittsburgh, PA 15240, USA; ^2^Center for Health Equity Research and Promotion, VA Pittsburgh Healthcare System, Pittsburgh, PA 15240, USA; ^3^Division of General Internal Medicine, Department of Medicine, University of Pittsburgh, Pittsburgh, PA 15261, USA; ^4^Department of Health Systems and Population Health, University of Washington School of Public Health, Seattle, WA 98195, USA; ^5^Health Services Research & Development (HSR&D) Center of Innovation for Veteran-Centered and Value-Driven Care, VA Puget Sound Health Care System, Seattle, WA 98108, USA

###### **Correspondence:** Rachel L. Bachrach (rachel.bachrach2@va.gov)

*Addiction Science & Clinical Practice* 2023, **18(Suppl 1)**: O36.

**Background:** Veterans Health Administration (VA) clinical guidelines stipulate that patients receive evidence-based alcohol-related care in PC (e.g., brief counseling interventions, pharmacotherapy), but many do not. We conducted qualitative interviews with clinical and Veteran stakeholders in one VA PC clinic to understand barriers and facilitators and tailor an implementation support intervention to help improve care.

**Materials and methods:** We interviewed 10 PC stakeholders (e.g., physicians, pharmacists) about: (1) experiences with and thoughts about providing alcohol-related care; and (2) feedback regarding the planned implementation support intervention. We then interviewed a purposive sample of Veterans with a history of unhealthy alcohol use (N = 22; ages‚â•18) seeking care at a northeastern VA PC clinic. Interviews assessed experiences in PC, alcohol/substance use treatment history, and ideas for improvement. Transcripts were analyzed using a rapid qualitative approach, which summarized interviews to extract key points and relevant themes.

**Results:** PC interviews revealed the following top barriers: variability in clinicians’ knowledge and confidence in defining unhealthy alcohol use and providing evidence-based care; logistical issues triaging/referring patients to behavioral health; competing clinical priorities (e.g., diabetes); and varying PC leadership support. Facilitators included belief in alcohol-related intervention in PC; identification of clinical champions; and support for implementation ideas presented (e.g., audit and feedback). Veteran interviews [45.5% female; Mage = 60.2; 41.0% Black; average AUDIT-C = 4.23 (range: 0–11)] revealed the following themes: overall positive experiences in PC, varying experience with and desire for alcohol-related care, desire for shared decision-making, and willingness to meet with other PC providers besides physicians to improve alcohol care.

**Conclusions:** Multidisciplinary providers and Veterans perspectives on delivering/receiving PC-based alcohol-related care supported development of a tailored multilevel implementation intervention that capitalizes on facilitators and minimizes barriers. PC providers should continue building compassionate relationships with Veterans and offer repeated non-judgmental evidence-based advice and treatment options, from a shared decision-making approach, regarding alcohol use.

### O37 SBIRT in non-medical settings for clients with substance use problems evaluation of its effectiveness

#### Fatima Abiola Popoola^1*^, Olibamoyo Olushola^2^, Ola Bolanle Adeyemi^2^

##### ^1^National Drug Law Enforcement Agency, Abuja, Nigeria; ^2^Department of Behavioural Medicine, Lagos State University College of Medicine Ikeja, Lagos, Nigeria

###### **Correspondence:** Fatima Abiola Popoola (abiolaodus9@gmail.com)

*Addiction Science & Clinical Practice* 2023, **18(Suppl 1)**: O37

**Background:** Screening, brief intervention, and referral to treatment (SBIRT) is an evidence-based practice that has been shown to reduce alcohol and drug use in healthcare and other settings, but there is a paucity of research on the effectiveness of SBIRT in a non-medical setting. These populations have high rates of substance use but have limited access to interventions.

**Materials and methods:** A simple random sampling technique was used for this research, 75 participants were selected from a pool of 150 users, referred mainly from criminal justice, place of work, and schools. Using the ASSIST, the intervention assessed the risk level for illicit drug use by participants and provided those who were at low or medium risk with a brief intervention and referred those at high risk to intensive treatment. RCQ was used to assess the level of motivation of participants. The intervention was given at baseline and 3 months following baseline intervention. Assessments were carried out at baseline and 6 months. Using interviews and records data from baseline and 6-month, analyses compared the differences. All analyses were set at 95% CI, p < 0.05, and were carried out by SPSS 22.0.

**Results:** We found that the risk of harmful use of cannabis, prescription opioids, and sedatives reduced significantly between baseline and 6-months so also were their mean ASSIST scores. Furthermore, participants have a statistically significant better level of motivation to stop the use of cannabis, prescription opioids, and sedatives between baseline and 6-months. Although participants have reduced risks of harmful use of solvent, the differences between baseline and 6-months were not significant.

**Conclusions:** This study has illustrated that the use of screening and the administration of brief interventions for reducing the harmful risk and improving the level of motivation to stop substance use in a non-medical setting can be feasible and effective.

### O38 Assessment by the JT method of brief intervention applied to Brazilian workers with a score of 8 or more in the audit

#### Sandra Regina Chalela Ayub^1*^, Raul Aragão Martins^2^

##### ^1^Fatec Catanduva-SP, Brasil; ^2^Departamento Educação, UNESP, Marília, Brasil

###### **Correspondence:** Sandra Regina Chalela Ayub (sandrachalela@gmail.com)

*Addiction Science & Clinical Practice* 2023, **18(Suppl 1)**: O38

**Background:** Alcohol Dependence Syndrome (ADS) is a disease worldwide considered a public health problem. Affects the worker's performance and the work environment due to the consequences of abusive use of alcohol, causing a drop in productivity and quality at work, as well as absences during the period of their journey; changes in personal habits, poor relationships with colleagues; accidents at work, among other vulnerabilities. This research aimed to evaluate, using the JT Method, the effect of a Brief Intervention (BI), aimed at returning to abstinence or at least to moderation, applied to workers who scored 8 or more in the AUDIT.

**Methods:** The research was carried out in four stages: initial survey (LI), interview, application of the BI and follow-up after six months. In LI, 229 workers participated, of which 78 (34.1%) reached the cut-off score (8 or more points). In the second stage, the results were confirmed with the Alcohol Dependence Scale—ADS and these workers were invited to participate in the IB, which was accepted by 46 of them. In the third stage, the IB was performed. In the follow-up session, the fourth stage of the research, the AUDIT was applied, after 6 months of the IB, in 23 participants who were located. Data analysis by the JT Method can result in four possibilities: positive maintenance, positive change, no change and negative change. The first case, positive maintenance, would be for participants who had a cutoff score below 8 points and would remain there, but who were not evaluated in this study.

**Results:** Results show that 14 (60.9%) participants had positive change and nine had no change. These results were satisfactory for the applied BI and can contribute to the implementation of programs aimed at workers' health using the IB, which is easy to apply, fast, low cost and effective.

### O39 Communities in Charge of Alcohol: Building capacity in alcohol screening and brief interventions through asset based community development

#### Elizabeth J Burns*, Cathy Ure, Penny Cook

##### School of Health & Society, University of Salford, Salford, M6 6PU, UK

###### **Correspondence:** Elizabeth J Burns (e.j.burns@salford.ac.uk)

*Addiction Science & Clinical Practice* 2023, **18(Suppl 1)**: O39

**Background:** Communities in Charge of Alcohol (CICA) takes an asset-based community development approach to build capacity in alcohol screening and brief interventions in the North West of England. Lay people volunteered to become an 'Alcohol Health Champion' (AHC) by attending a Royal Society of Public Health (RSPH) training course to equip them with the knowledge and skills to use AUDIT-C and provide opportunistic informal brief advice. An additional half day course was provided on how to navigate the alcohol licensing process in England.

**Methods:** The process evaluation explored the coordination, recruitment, training delivery, and volunteer activity of AHCs. Data collection included training registers, pre- and post-training questionnaires, reflective diaries, interviews with key stakeholders and interviews with Alcohol Health Champions at 3 months and 12 months. Focus groups were carried out with community members after the first year. Data were analysed using descriptive statistics, framework analysis and thematic analysis.

**Results:** In total, seven areas coordinated the delivery of the CICA programme for 12 months, training 123 people as AHCs including 95 lay volunteers. Having brief conversations about alcohol was shown to be the predominant activity of AHCs. Focus groups highlighted the felt needs of community representatives were orientated towards improving the local alcohol treatment system to provide better responses to alcohol dependence.

**Conclusion**: CICA was successful in training lay people in health-deprived areas, providing them with a qualification and confidence to provide brief advice. The felt needs of AHCs and community representatives highlighted the importance of having integrated local treatment systems for alcohol misuse in place when building capacity in early intervention.

### O40 Piloting a strategy to screen and detect risky drinking of alcohol in the community pharmacies of Catalonia

#### Joan Colom Ferran^1*^, Jorge Palacio-Vieira^1^, Berta Torres-Novellas^2^, Toni Veciana^2^, Lidia Segura-García^1^, Estela Díaz^1^, Gemma Galofré Pomés^3^

##### ^1^Program on Substance Abuse, Public Health Agency of Catalonia, Health Department of the Government of Catalonia, Roc Boronat 81-95, 08005, Barcelona, Spain; ^2^Catalan Council of Pharmacists’ Associations. Girona 64-66, 08009, Barcelona, Spain; ^3^Tarragona Pharmacists Association, Enric d'Ossó, 1, 43005, Tarragona, Spain

###### **Correspondence: **Joan Colom Ferran (joan.colom@gencat.cat)

*Addiction Science & Clinical Practice* 2023, **18(Suppl 1)**: O40

**Background:** Community pharmacists (CP) may play an important role in the identification and management of alcohol consumption at the population level. The objective of this study is to show the results of piloting a novel strategy addressed to the identification and brief intervention of alcohol problems among the community pharmacies users.

**Materials and methods:** A total of 36 CP of Tarragona (Catalonia) were invited to screen risky drinkers using the AUDIT-C (cut-off points of 5 or more for men and 4 or more for women) specifically among those who attended their pharmacies to acquire medications that interact with alcohol, including Benzodiazepines, Acenocumarol, Levothyroxine, Carbimazole, pregnancy test or emergency contraceptive pills. In addition, three options of brief intervention were used, face-to-face oral advise, leaflet council and referral to primary healthcare units.

**Results:** During 45 days of piloting, a total of 17 CP (47%) screened 173 eligible users, 68% of them were women, the mean age was 53.4 years (50.2 among women and 60.1 among men) and 45% of users screened acquired Benzodiazepines. Among those who accepted being screened (n = 149), 20% were non-drinkers, and among drinkers 43% were considered risky drinkers. A total of 62% of drinkers received a face-to-face oral intervention, 88% received leaflets and less than 1% were referred to primary health care units. The average time reported for screening and brief intervention was less than 5 min in the 52% of cases, between 5 and 10 min in 36% of the cases and more than 10 min in 12% of the cases.

**Conclusions:** Screening and brief intervention on alcohol are both feasible at the community pharmacies. Ensuring that time does not exceed 5 min, targeting risky drinkers and those who acquire medications that interact with alcohol could easy and ensure the implementation of SBI other regions of Catalonia.

### O41 Opportunities for screening, brief interventions, and treatment of alcohol use disorders in low-income countries: Lessons from the SAFER program in Uganda

#### David Kalema*

##### Uganda Alcohol Policy Alliance, Uganda

###### **Correspondence:** David Kalema (kalemdav@gmail.com)

*Addiction Science & Clinical Practice* 2023, **18(Suppl 1)**: O41

**Background:** SAFER represents 5 effective and cost-effective alcohol control interventions including (1) Strengthening restrictions on alcohol availability, (2) Advancement of drink-driving countermeasures, 3) Facilitating Screening, Brief Interventions and Treatment (SBIT), (4) Enforcement of bans or comprehensive restrictions on advertising, sponsorship and promotion and (5) Raising prices on alcohol through excise taxes and pricing policies. Uganda is the first country to partner with the World Health Organization (WHO) led SAFER Initiative and this collaboration has the potential to provide leadership and lessons to other governments and advocates seeking to reduce the health, social and economic impacts of harmful use of alcohol. SBIT was identified among the priority SAFER interventions with high need and high feasibility in Uganda.

**Materials and methods:** This presentation focuses on the SAFER desk-based review and joint WHO and Ministry of Health joint programming mission in regards to SBIT in Uganda. The report analyses potential facilitators and barriers to Uganda’s response to the harmful use of alcohol and shall provide an update on the implementation of the suggested 24-month (effective January 2022) multi-sectoral roadmap to scale up SBIT services in Uganda.

**Results:** Barriers/Opportunities; Although established government and private facilities and peer support groups offer services for AUD treatment, they are few and segmented, with a general lack of specialized professionals. Besides, the coverage of AUDs in the Uganda Clinical Guidelines is insufficient.

**Conclusions:** Recommendations for enhancing SBIT include training regional focal persons in hospitals and schools; facilitating peer support groups at regional and community levels; establishing an accreditation system for AUD services and an inventory of private treatment centers; integrating AUD indicators/statistics in the National Health Information Management System; and promoting SBIT for Uganda Police Force members.

### O42 Training of trainers on screening and brief intervention and referral to treatment (SBIRT) for tobacco use

#### Marianne Hochet^1^*, Nicolas Bonnet^1^

##### ^1^RESPADD (French Network for addiction prevention), Paris, France

###### **Correspondence:** Marianne Hochet (marianne.hochet@respadd.org)

*Addiction Science & Clinical Practice* 2023, **18(Suppl 1)**: O42.

**Background:** The prevalence of smoking is still very high in France despite all the public health actions led during past years. 25% of people aged between 18 and 75 years old are daily smokers in France. One action in France is to encourage and support hospitals and health services in becoming tobacco-free. In order for this strategy to be efficient, health care professionals must be trained to screen smokers and support them quitting. Thus, the French addiction prevention network, RESPADD, which is the national coordinator of this important strategy, is implementing training of trainers to quickly disseminate knowledge and know-how.

**Materials and methods:** With the help of each regional health agencies, we have set up at least one training of trainers on SBIRT for tobacco use in all French regions during the past three years. For each session, around 20 persons were trained during two days on tobacco basic knowledge, SBIRT and how to train colleagues on this topic and thus spread information. Participants were mainly healthcare professionals, with the ability to prescribe nicotine replacement therapy like nurses and doctors or not allowed to prescribe but to discuss tobacco consumption with patients. Several tobacco-free hospital project coordinators were also trained.

**Results:** In three years, more than 200 new trainers were trained on the previous mentioned topics and are now able to spread knowledge and know-how in their own health structure but also elsewhere. Each region has now its own trainers on SBIRT for tobacco use.

**Conclusions:** People are willing to learn more on tobacco issues and to spread knowledge as trainers which is part of the evidence-based program of tobacco-free hospitals.

### O43 Nursing students: experience with the training and implementation of a brief alcohol motivational interviewing program

#### Maria Lavilla Gracia^1^*, Navidad Canga Armayor^1^, Maria Pueyo Garrigues^1^

##### ^1^Community, Maternity and Pediatric Nursing Department, University of Navarra, Pamplona, Spain

###### **Correspondence:**Maria Lavilla Gracia (mlavilla@unav.es)

*Addiction Science & Clinical Practice* 2023, **18(Suppl 1)**: O43

**Background:** Peer-led brief motivational interventions in the university setting are promising for reducing alcohol consumption and related negative consequences among undergraduates. Knowing peer counsellors‚Äô competence and their experience in conducting this kind of intervention is important to improve their training; unfortunately, evidence on this topic is scarce.

**Aim:** To evaluate nursing students’ competence and their experience in leading a brief alcohol-focused motivational interviewing with their peers.

**Materials and methods:** A mixed-method study was undertaken with 21 peer counsellors. Competency in motivational interviewing and alcohol-related content was evaluated through the Peer Proficiency Assessment instrument and a checklist coding two videotaped interventions by each participant. Experiences were explored through three focus groups (they were asked about their strengths, weaknesses, benefits, and challenges of being students who do the interviews), and content analysis was undertaken.

**Results:** In general, nursing students achieved proficiency in motivational interviewing by asking more open-ended questions and complex reflections. All of them covered most of the alcohol-related topics that should come up during the interview. Regarding their experiences, they found being a peer as a strength because of the confidence and openness that students have by sharing common experiences. On the other hand, the most common challenges where the lack of self-confidence and experience, conflict of role between being a peer and being professional and they struggle with the preconceptions about peer status. Most peer counsellors agree that they achieved the goal of making the student reflect on their alcohol consumption.

**Conclusions:** Nursing students who receive training in alcohol-focused motivational interviewing improve their skills. In addition, they feel satisfied when conducting this intervention with their peers to raise awareness about alcohol consumption. However, before conducting an interview with an alcohol student user, they need to do more practice during the training.

### O44 A dose–response analysis of adolescent SBIRT education for health professionals: navigating a post-COVID landscape

#### Weiwei Liu^1*^, Jackie Sheridan-Johnson^2^, Hildie Cohen^2^, Tracy L. McPherson^2^

##### ^1^Public Health, NORC at the University of Chicago, Bethesda, MD 20814, USA; ^2^Public Health, NORC at the University of Chicago, Chicago, IL 60603, USA

###### **Correspondence:** Weiwei Liu (liu-weiwei@norc.org)

*Addiction Science & Clinical Practice* 2023, **18(Suppl 1)**: O44

**Background:** The screening, brief intervention, and referral to treatment (SBIRT) model is effective in preventing and reducing substance use among youth. NORC at the University of Chicago, along with leading professional education experts, developed and tested an adolescent SBIRT curriculum for use in nursing, social work, and interprofessional education. In prior publications, we demonstrated that this training is effective in preparing health professionals to implement adolescent SBIRT. Less understood is how the effectiveness of such training may vary as a function of setting and dosage. This study evaluated the impact of implementation setting (online, in-person, hybrid) and dosage (hours of SBIRT) on students’ attitudes; perceived readiness, confidence, competence; knowledge, and skills.

**Materials and methods:** Students completed a pre-training survey, adolescent SBIRT education, and post-training survey. The sample included 33 schools with 1,646 students. In addition to analysis of the overall sample, we stratified analyses by graduate and undergraduate students. We also stratified by pre- and post-COVID to account for the ubiquitous shift to online learning and changes in online training delivery. OLS regression models were conducted to evaluate pre-post changes by implementation setting and dosage, adjusting for student’s level and prior training.

**Results:** For undergraduate students, longer SBIRT training was positively associated with improvement in confidence and readiness; this effect was not significant for graduate students. Pre-COVID, online learning was associated with less improvement on competence, confidence, and readiness compared to in-person training. This reversed post-COVID, with online learning associated with greater improvement than in-person learning.

**Conclusions:** Findings suggest that longer training may lead to greater improvement in outcome measures for certain subgroups. The drastic and rapid shift to online learning introduced by COVID-19 did not negatively impact effectiveness of SBIRT education and may have improved online learning tools that can be utilized for adolescent SBIRT training in the future.

### O45 Association between previous emotional state and drinking risk levels in young adults

#### Maria Lucia Oliveira de Souza Formigoni^1^, Giovanna T Petucco^1^, Professor Claudia B Mello^1^

##### ^1^Departamento de Psicobiologia, Escola Paulista de Medicina, Universidade Federal de São Paulo, São Paulo, Cep 04023062, Brazil

###### **Correspondence:** Maria Lucia Oliveira de Souza Formigoni (mlosformigoni@unifesp.br)

*Addiction Science & Clinical Practice* 2023, **18(Suppl 1)**: O45

**Background:** When carrying out brief interventions for alcohol misusers, it is important to understand the situations that trigger excessive consumption. Alcohol use in social gatherings may involve consumption to deal with positive or negative emotions or to facilitate the creation of social bonds. In this study, we investigated the association between antecedent emotional situations and the classification of young people according to their drinking risk levels.

**Materials and methods:** We evaluated 122 volunteers, aged between 18 and 25 years old, regarding their alcohol consumption and associated problems (Alcohol Use Disorders Identification Test-AUDIT scores), drinking risk situations (Inventory of Drinking Situations/IDS-42), family history of psychoactive substances related problems and sociodemographic characteristics. We tested the association between IDS-42 situations and AUDIT scores using regression analysis and compared IDS-42 items scores between low-risk and at-risk/suggestive of dependence groups by Students t test.

**Results:** Out of the 122 volunteers, 91 were female and 31 male; 79 participants were classified by AUDIT in Zone 1 (low risk) and 43 in the at-risk/ suggestive of dependence (zones II-IV). Linear regression analyses indicated higher AUDIT scores were associated with: drinking when they presented unpleasant emotions (p < 0.001); test of personal control (p = 0.01) or when they have pleasant times with others (p = 0.006). The IDS-42 item (I drank heavily) When I wanted to feel closer to someone I liked stands out, with significantly higher scores in the high-risk/suggested of dependence group (p = 0.001).

**Conclusions:** Detecting high-risk drinking situations is essential to plan adequate Brief Interventions. Difficulties in the ability to relate to other people is one of the reasons that lead young people to misuse alcohol. In the next phase of the study, we will test the associations between social cognition skills and drinking related problems.

### O46 Development of a mobile application for screening, brief intervention and referral of adolescents and young adults with substance use disorders

#### Maria Lucia Oliveira de Souza-Formigoni^1^, Richard A. Reichert^1^, Denise De Micheli^1^, Matheus Sabino de Souza^3^, Adriana Gomes Alves^3^, Paulo Bandiera Paiva^2^, Antonio Carlos da Silva Junior^2^, Anne H Berman^4^

##### ^1^Departamento de Psicobiologia, Escola Paulista de Medicina, Universidade Federal de São Paulo, São Paulo, Brazil Cep 04023062; ^2^Departamento de Informática em Saúde, Escola Paulista de Medicina, Universidade Federal de São Paulo, São Paulo, Brazil Cep 04023062; ^3^Universidade do Vale do Itajaí, Itajaí, Santa Catarina, Brazil; ^4^Department of Psychology, Uppsala University, Uppsala, Sweden

###### **Correspondence:** Maria Lucia Oliveira de Souza-Formigoni (mlosformigoni@unifesp.br)

*Addiction Science & Clinical Practice* 2023, **18(Suppl 1)**: O46

**Background:** Alcohol and other drug use disorders among youth are a public health concern worldwide. The dissemination of Screening and Brief Interventions (SBI) directed to adolescents and young adults is important to mitigate the potential social and health harms related to drugs misuse.

**Materials and methods:** The objectives of this project were: a) to perform the cross-cultural adaptation and validation of the DUDIT-E (Drug Use Disorders Identification Test Extended) screening scale to be used in Brazil; b) to develop a digital application to be used by health professionals or social workers including SBI and referral of adolescents and young adults with drug use disorders. This interdisciplinary and multicenter project has been carried out in Brazil at the Deparments of Psychobiology and Informatics in Health of Universidade Federal de São Paulo (UNIFESP) in partnership with researchers from the Universidade do Vale do Itaja (UNIVALI) and Uppsale University (Sweden), integrating knowledge from the areas of health and computer science.

**Results:** The (DUDIT-E) was translated into Portuguese, back-translated and approved by the authors of the original version, then adapted for online use. The app for mobile devices (TrIE-AD) includes: a) the online version of the DUDIT-E to assess the patterns of use of psychoactive substances, the positive and negative perceptions on drug use and the level of motivation and readiness to change behavior; (b) guided BI based on FRAMES (Feedback, Responsibility, Advice, Menu of options, Empathy, and Self-efficacy) principles and on the answers to DUDIT-E; (c) links to referral to specialized services, and (d) links to complementary materials for training of professionals.

**Conclusions:** In the next phase of the study the adherence of professionals to the app will be evaluated and its use is expected to improve SBI dissemination for the reduction of problems associated with drug use by young people.

### O47 Brief parent interventions: Parents needs and recommendations for the prevention of alcohol intoxications among teenagers

#### Silke Diestelkamp^*^, Rainer Thomasius

##### German Center for Addiction Research in Childhood and Adolescence, University Medical Center Hamburg-Eppendorf, Martinistr. 52, D-20246 Hamburg, Germany

###### **Correspondence:** Silke Diestelkamp (s.diestelkamp@uke.de)

*Addiction Science & Clinical Practice* 2023, **18(Suppl 1)**: O47

**Background:** In Germany, around 20.000 children and adolescents are beeing treated for acute alcohol intoxication every year. Every single alcohol intoxaction is associated with an elevated risk of experiencing a number of negative consequences, such as inju-ry, violence or sexual assault. Parent interventions may contribute to preventing a-cute alcohol intoxications in children and adolscents. The current study aimed at identifying circumstances which lead to alcohol intoxications among teenagers as well as parents needs and recommendations for preventive parent interventions.

**Materials and methods:** N = 201 children and adolescents who had received a brief intervention following acute alcohol intoxication participated in the quantitative study on circumstances which lead to the alcohol intoxication. Additionally, in-depth interviews with N = 9 caregivers and N = 8 children and adolescents were conducted. The semi-structured telephone interviews assessed rules regarding alcohol use before and after the in-toxication, consequences of non-compliance with rules and needs and recommen-dations for preventive parent interventions.

**Results:** Five of nine parents reported to have had specified alcohol-related rules with their child before the intoxication. Six of nine parents introduced new rules after the into-xication with a focus on enhanced monitoring, more communication about their children’s alcohol use and specific rules, e.g. related to participation in drinking ga-mes. Five parents introduced new consequences for non-compliance with alcohol-related rules after the intoxication. Consequences for non-compliance can be grouped into either intensified communication or sanctions. Parents articulated needs to reflect on advantages and disadvantages of rules containing prohibitions and to exchange experiences with other parents. Six of nine parents recommend to talk to children about alcohol use before they start consuming.

**Conclusions:** Parent interventions should take place before children start consuming alcohol and should include opportunities to reflect on alcohol-related rules and consequences of non-compliance as well as give parents the opportunity to exchange opinions and experiences.

### O48 Comparison of substance use screening instruments and time frames among adolescents in rural health clinics

#### Jan Gryczynski^1*^, Laura Monico^1^, Kevin O'Grady^2^, Mishka Terplan^1^, Shannon Gwin Mitchell^1^

##### ^1^Friends Research Institute, Baltimore, MD, USA; ^2^University of Maryland, College Park, MD, USA

###### **Correspondence:** Jan Gryczynski (jgryczynski@friendsresearch.org)

*Addiction Science & Clinical Practice* 2023, **18(Suppl 1)**: O48

**Background:** Initiation of substance use often occurs during adolescence. Many young people access primary care, which offers an opportunity for screening and brief intervention. Several self-report screening tools have been validated with adolescents that query behavior in different time frames.

**Methods:** This is a secondary analysis of baseline data from a multi-site, stepped wedge trial of the FaCES organizational change package to promote adoption of SBIRT services in adolescent primary care. The analysis sample includes N = 1119 adolescent patients ages 12–17 recruited from five rural primary care clinics in New Mexico and Tennessee, USA. Participants completed several self-report screening questionnaires (S2BI, BSTAD, ASSIST) at enrollment. We compared self-reported substance use at different time frames used by these measures (lifetime, past year, past 3-months, past 30-days) and examined their concordance using Cohen’s kappa. Differences in kappas were tested using Gwet’s procedure for comparing correlated kappas.

**Results:** Tobacco, alcohol, and cannabis were the most commonly reported substances, with lifetime use (ASSIST) reported by 24%, 28%, and 19%, respectively. Rates of past year use (S2BI) of tobacco, alcohol, and cannabis were 18%, 19%, and 15%, respectively, while past 30-day use (BSTAD) was reported by 12%, 8%, and 8%, respectively. Comparing lifetime (ASSIST) to past year (S2BI), 3-month (ASSIST), and 30-day (BSTAD) disclosure, kappa concordance values were 0.80, 0.68, and 0.57 [tobacco]; 0.75, 0.56, 0.33 [alcohol]; and 0.86, 0.74, 0.54 [cannabis], respectively. As expected, there was a significant decline in degree of agreement (i.e., magnitude of kappas) with widening windows of time (ps < 0.05).

**Conclusions:** Time frame is an important consideration in substance use screening. Querying substance use over more extended time horizons (lifetime, past year) yields higher rates of disclosure and identification (with potential to reinforce recent abstinence), but historical measures may not reflect recent behavior even among adolescents.

### O49 Using health utility to set drinking targets for alcohol brief interventions

#### Jeremy W. Bray^1*^, Arnie Aldridge^2^, Carolina Barbosa^3^, Abraham Gebreselassie^1^, Collin Labutte^1^, Eve Wittenberg^4^

##### ^1^Department of Economics, UNC Greensboro, Greensboro, NC, USA; ^2^RTI International, RTP, NC, USA; ^3^RTI International, Chicago, IL, USA; ^4^Center for Health Decision Science, Harvard T.H. Chan School of Public Health, Boston, MA, USA

###### **Correspondence:** Jeremy W. Bray (jwbray@uncg.edu)

*Addiction Science & Clinical Practice* 2023, **18(Suppl 1)**: O49

**Background:** Low-risk drinking guidelines are often used to set drinking targets for alcohol brief interventions, but their morbidity and mortality focus ignore the quality of life of the drinker. To better inform low-risk drinking guidelines, we estimate the relationship between specific drinking behaviors and health utility, a measure of health-related quality that is the basis for quality adjusted life years (QALYs).

**Materials and methods:** We use data from the US nationally representative National Epidemiologic Survey on Alcohol and Related Conditions-III (NESARC-III) dataset. We modeled individuals’ health utility as a quadratic function of typical quantity consumed, typical frequency of consuming that amount, maximum quantity consumed in a single occasion, and frequency of consuming that amount while limiting our analyses to the target population for most alcohol brief interventions‚Äîcurrent drinkers with no history of AUD.

**Results:** For typical frequency and quantity, utility increased with frequency but decreased with quantity, suggesting that utility was maximized by a typical consumption pattern of 1 drink per occasion, 5 days per week. For maximum quantity and frequency, however, utility decreased with frequency and increased quantity, suggesting utility was maximized when the largest amount consumed on a single occasion was about 3 drinks no more than 1 day per year. Taking both patterns into account, our estimates suggest that overall utility is maximized by consuming 1 drink per occasion on about 4 days per week.

**Conclusions:** When the health utility of drinkers is considered, the optimal level of drinking is less than that recommend by many low-risk drinking guidelines, suggesting that alcohol brief interventions should target lower consumption levels than they currently do. Furthermore, because health utility is the basis for calculating QALYs, such intervention targets may result in more QALYs gained and thereby increase the cost-effectiveness of brief interventions.

### O50 Incorporating universal opioid use disorder screening into primary care: experiences from a national cohort of U.S. clinics

#### Emily C. Williams^1,2*^, Elizabeth J. Austin^1^, Elsa S. Briggs^1^, Lori Ferro^3^, Paul Barry^4^, Ashley Heald^4^, Geoff M. Curran^5^, Andrew Saxon^3,6^, John Fortney^2,3^, Anna D. Ratzliff^3,6^

##### ^1^Department of Health Systems and Population Health, School of Public Health University of Washington, Seattle, WA, USA; ^2^Center of Innovation for Veteran-Centered and Value-Driven Care, Health Services Research & Development, VA Puget Sound, Seattle, WA, USA; ^3^Department of Psychiatry and Behavioral Sciences, School of Medicine, University of Washington, Seattle, WA, USA; ^4^Advancing Integrated Mental Health Solutions (AIMS) Center, University of Washington, Seattle, WA, USA; ^5^Departments of Pharmacy Practice and Psychiatry, University of Arkansas for Medical Sciences, Little Rock AR; Central Arkansas Veterans Health Care System, Little Rock, AR, USA; ^6^Center of Excellence in Substance Addiction Treatment and Education, VA Puget Sound, Seattle, WA, USA

###### **Correspondence:** Emily C. Williams (emwilli@uw.edu)

*Addiction Science & Clinical Practice* 2023, **18(Suppl 1)**: O49

**Background:** In response to rising incidence of and associated mortality with opioid use disorder (OUD) and evidence that identification followed directly by treatment can support OUD reduction, the U.S. Preventive Services Taskforce recommends routine screening for OUD, in primary care settings. Yet little is known about the barriers primary care teams face when trying to implement OUD screening into practice. In a randomized trial to integrate OUD treatment alongside collaborative care for behavioral health, we supported 10 U.S. primary care clinics in implementing OUD screening and documented early implementation experiences using formative evaluation.

**Materials and methods:** Trained qualitative researchers took detailed observation notes at implementation meetings with individual clinics and regular debriefings with practice facilitators (n = 149 meetings). Fieldnotes were analyzed weekly using a Rapid Assessment Process guided by the Consolidated Framework for Implementation Research. After clinics launched OUD screening, we conducted a structured fidelity assessment with each site to systematically assess clinic experiences. Data from fieldnotes and structured assessments were combined into a matrix to compare across clinics and identify common patterns and cross-cutting themes. Resultant themes from early implementation were iteratively reviewed with the study team.

**Results:** While all clinics had the goal of implementing population-based OUD screening, clinics experienced barriers across multiple domains, including: (1) challenges identifying which patients to screen, (2) complexity of the screening recommended tool, (3) staff discomfort, (4) workflow barriers that decreased follow-up to positive screening/referral to treatment, (5) staffing shortages and turnover, (6) discouragement from low screening yield, and (7) stigma. Promising implementation strategies included: a more universal screening approach, health information technology (HIT), audit and feedback, and repeated staff trainings.

**Conclusions:** Implementing OUD screening in diverse primary care clinics was challenging. Implementation strategies that standardize workflows via HIT, decrease stigma, and increase staff knowledge and confidence regarding OUD care may increase feasibility.

### O51 PROUD trial main results: a pragmatic implementation trial testing the Massachusetts model of nurse collaborative care for opioid use disorder

#### Kathy Bradley^1^, Jennifer F. Bobb^1^, Abigail G. Matthews^2^, Denise M. Boudreau^1^, Paige D. Wartko^1^, Jennifer McCormack^2^, David S. Liu^2^, Cynthia I. Campbell^3^, Amy K. Lee^1^, Jeffrey H. Samet^4^, Colleen T. Labelle^4^, Megan Addis^1^, Onchee Yu^1^, Abisola Idu^1^, Hongxiang (David) Qiu^5^, Noorie Hyun^1^, Joseph E. Glass^1^, Ryan Caldeiro^6^, Julia Arnsten^7^, Chinazo Cunningham^7^, Jordan M. Braciszewski^8^, Amy Loree^8^, Angela Stotts^9^, Mohammad Zare^9^, José Szapocznik^10^, Viviana Horigian^10^, Mark Murphy^11^, Andrew J. Saxon^12^

##### ^1^Kaiser Permanente Washington Health Research Institute, Seattle, WA 98101, USA; ^2^National Institute on Drug Abuse, Bethesda, MD, USA; ^3^RI Center for Community Health and Evaluation, Kaiser Permanente Southern California, Oakland, CA, USA; ^4^Boston Medical Center, Boston, MA, USA; ^5^University of Washington, Seattle, WA, USA; ^6^Kaiser Permanente Washington, Everett, WA, USA; ^7^Montefiore Medical Center, New York, NY, USA; ^8^Henry Ford Health System, Detroit, MI, USA; ^9^Harris Health System, Houston, TX, USA; ^10^Miller School of Medicine, University of Miami, Miami, FL, USA; ^11^MultiCare, Tacoma, WA, USA; ^12^Veterans Affairs Puget Sound Health Care System, Seattle, WA, USA

###### **Correspondence:** Kathy Bradley (katharine.a.bradley@kp.org)

*Addiction Science & Clinical Practice* 2023, **18(Suppl 1)**: O51

**Background:** Evidence-based treatment for opioid use disorder (OUD) includes two medications that can be prescribed in primary care (PC): buprenorphine and injectable naltrexone (NTX). Despite recommendations to treat OUD in PC, few PC practices do so. The PRimary care Opioid Use Disorders Treatment (PROUD) Trial was a pragmatic implementation trial (NCT03407638) that evaluated whether implementation of the Massachusetts Model of nurse care management increased medication treatment for OUD in PC (primary objective) and decreased acute care utilization among patients with OUD pre-randomization (powered secondary objective).

**Materials and methods:** PROUD was conducted in 6 U.S. health systems (3/1/2018‚Äì2/29/2020). We randomized two PC clinics in each health system (n = 12) to PROUD Intervention or Usual Care. All quantitative data were obtained from secondary electronic health records and insurance claims with waivers of consent and HIPAA authorization. The primary outcome was a clinic-level measure of patient-years of OUD treatment with buprenorphine or NTX. The secondary outcome was a patient-level measure of days of acute care utilization. The Trial tested one-sided hypotheses: whether the intervention 1) increased OUD treatment and 2) decreased acute care utilization (Œ ±  = 0.05).

**Results:** Intervention and Usual Care clinics included 130,623 and 159,459 patients respectively. Intervention clinics provided 8.2 (95% CI: 5.39, ‚àû) more patient-years of OUD treatment (p = 0.002), per 10,000 PC patients post randomization, compared with Usual Care. These benefits were largely driven by 2 health systems. The Intervention increased treatment in men more than women (p for interaction = 0.047). Days of acute care utilization did not differ between Trial arms (RR 1.16; 95% CI: 0.47, 2.92; p = 0.70).

**Conclusion:** The PROUD Intervention implementing the Massachusetts Model of nurse care management increased PC OUD treatment. Additional improvements or interventions will be needed to increase OUD treatment consistently across systems and among women, as well as decrease days of acute care utilization.

### O52 Successful implementation of substance use screening in rural federally qualified health centres identified high rates of unhealthy alcohol, cannabis, and tobacco use

#### Jennifer McNeely^1*^, Bethany McLeman^2^, Trip Gardner^3^, Noah Nesin^3^, Sarah Farkas^4^, Aimee Wahle^5^, Seth Pitts^5^, Margaret Kline^5^, Jacquie King^5^, Carmen Rosa^6^, Lisa Marsch^2^, John Rotrosen^4^, Leah Hamilton^7^

##### ^1^Department of Population Health, New York University Grossman School of Medicine, New York, NY 10016, USA; ^2^Center for Technology and Behavioral Health, Geisel School of Medicine at Dartmouth College, Lebanon, NH 03766, USA; ^3^Penobscot Community Health Center, Bangor, ME 04401, USA; ^4^Department of Psychiatry, New York University Grossman School of Medicine, New York, NY 10016, USA; ^5^The Emmes Company, Rockville, MD 20850, USA; ^6^National Institutes of Health, National Institute on Drug Abuse, Bethesda, MD  20852, USA; ^7^Kaiser Permanente Washington Health Research Institute, Seattle, WA 98101, USA

###### **Correspondence:** Jennifer McNeely (jennifer.mcneely@nyulangone.org)

*Addiction Science & Clinical Practice* 2023, **18(Suppl 1)**: O52.

**Background:** Screening for substance use in rural primary care clinics faces unique challenges due to limited resources, high patient volumes, and multiple demands on providers. To explore the potential for electronic health record (EHR)-integrated screening, we conducted an implementation feasibility study with a rural federally-qualified health center (FQHC) in Maine. This was an ancillary study to a NIDA Clinical Trials Network study of screening in urban clinics (CTN-0062).

**Methods:** Researchers worked with stakeholders from 3 FQHC clinics to define and implement their optimal screening approach. Clinics used the TAPS Tool, completed on tablets in the waiting room; results were immediately recorded in the EHR. Adults presenting for annual preventive care visits were eligible for screening. Data were collected between 11/1/2018–5/5/2020, and analyzed for 12 months following implementation at each clinic to assess screening rates and prevalence of reported unhealthy substance use.

**Results:** Screening was completed by 3,749 patients, representing 93.4% of those eligible and 18.4% of all adult patients presenting for primary care visits. In 92.9% of cases, screening was self-administered. Current unhealthy substance use (TAPS score 1 + for at least one substance) was identified in 1,219 patients (32.5% of those screened): 508 (13.6%) had unhealthy use of tobacco, 1064 (28.4%) alcohol, 383 (10.2%) cannabis, 11 (0.3%) illicit drugs, and 18 (0.5%) non-medical use of prescription drugs.

**Conclusion:** Self-administered EHR-integrated screening was feasible to implement and detected substantial alcohol, cannabis, and tobacco use in rural FQHC clinics. Rates of drug use (including cannabis) identified through screening were higher (10% vs. 0.3–1.0%) than in the parent study, possibly because the TAPS allows patients to report cannabis separately from other drugs in a cannabis-legal state. Future work may broaden the reach of screening by offering it at routine visits rather than restricting to annual preventive care, within these and other rural clinics.

### O53 Profile of Substance dependence with adult ADHD: a study from a tertiary care setting in India

#### Sneha Goyal^1*^, Paulomi M. Sudhir^1^, Vivek Benegal^2^, Keshav Kumar^1^, Urvakhsh M. Mehta^2^

##### ^1^Department of Clinical Psychology, NIMHANS, Bangalore; ^2^Department of Psychiatry; Head Centre for Addiction Medicine (CAM), NIMHANS, Bangalore, India

###### **Correspondence:** Sneha Goyal (sneha.goyal591@gmail.com)

*Addiction Science & Clinical Practice* 2023, **18(Suppl 1)**: O53

**Background:** One of the neurodevelopmental risk factors for substance dependence is adult ADHD. Individuals with alcohol use and ADHD are noted to have higher alcohol consumption, faster progression towards dependence, shorted abstinence periods, and early relapses. Comorbid alcohol use and ADHD may also be associated with greater number of Axis-I comorbidities. Individuals with Nicotine use and cannabis with comorbid ADHD are noted to have earlier age of onset, cognitive biases about substance-use, and greater use of substances for coping. Individuals with nicotine dependence and adult ADHD are also noted to have difficulty quitting and worse withdrawal symptoms. Indian literature has pointed towards high overlap between substance-dependence and ADHD. The present study aims to examine profile of adults with Substance-related and Addictive disorders with comorbid ADHD.

**Methods:** In the present study, 43 individuals with Substance-related and Addictive disorders and comorbid adult ADHD were recruited from a tertiary-care hospital setting in South India. Mean age of participants was 27 years, with 41 males and 2 females, belonging to middle socio-economic status. Data was analyzed for a pattern of substance use, severity of ADHD, nature of impulsivity, executive functions, socio-emotional factors, and functioning. Most common substances were noted to be alcohol, nicotine, and cannabis and one individual had Gambling disorder. Majority of the sample had one additional comorbid axis I diagnosis.

**Results:** The results indicated higher rate of dependence of nicotine and cannabis, and higher rate of abstinence with alcohol. The results also suggested moderately severe ADHD, high impulsivity, and poor cognitive-emotional-social functioning. The study has two major implications, 1) there is a need to screen for neurodevelopmental vulnerability factors like ADHD in population of adults with tobacco, alcohol, and cannabis use, or polysubstance use; and 2) early treatment of ADHD is likely to reduce the risk for developing substance use.

### O54 Targeted SBIRT and homelessness prevention intervention for emergency department patients with drug use or unhealthy alcohol use: a pilot feasibility study

#### Kelly Doran^1*^, Daniela Fazio^1^, Sara Zuiderveen^2^, Dana Guyet^2^, Andrea Reid^2^

##### ^1^Departments of Emergency Medicine and Population Health, NYU School of Medicine, New York, NY 10016, USA; ^2^Homelessness Prevention Administration, NYC Human Resources Administration, New York, NY 10007, USA

###### **Correspondence:** Kelly Doran (kelly.doran@nyulangone.org)

*Addiction Science & Clinical Practice* 2023, **18(Suppl 1)**: O54.

**Background:** Housing insecurity is commonly endorsed by emergency department (ED) patients, and is particularly prevalent among ED patients with drug or unhealthy alcohol use. We describe a pilot study of an intervention to simultaneously address substance use and homelessness risk among ED patients.

**Materials and methods:** We randomly approached patients at an urban public hospital ED to assess for eligibility. Eligible patients were adults, medically stable, not incarcerated, spoke English, screened positive for unhealthy alcohol or any drug use using single-item screening questions, and were not homeless but screened positive for risk of future homelessness using a previously-developed three-item homelessness risk screening tool (HRST). The study intervention consisted of: (1) brief counseling and referral to substance use treatment via a pre-existing ED program; (2) enhanced referral to Homebase, an evidence-based homelessness prevention program; (3) up to 3 troubleshooting phone calls to ensure participants accessed Homebase. Participants completed questionnaires at baseline and 6 months. The study was IRB approved.

**Results:** Of 2,183 patients screened, 51 were eligible; most screened negative on the HRST and thus were ineligible. Forty of 51 (78%) eligible patients participated; 1 later withdrew. Of the 32 (82%) participants reached at 6 months, most said it was very or extremely helpful talking to someone about their housing situation (n = 23, 72%) and receiving resources about substance use (n = 21, 66%) in the ED. Thirteen (41%) said their housing situation had improved in the past 6 months and 16 (50%) said it had not changed. Twenty (62.5%) had made contact with a Hombase office, 50% of whom said Homebase services were helpful. Thirty-one (97%) were satisfied with the study experience.

**Conclusions:** Our ED pilot intervention to address homelessness risk and substance use was feasible and well-received. Addressing homelessness risk in tandem with substance use interventions in the ED warrants future study.

### O55 Understanding the acceptability and experiences of screening and brief interventions for problematic alcohol consumption in people with co-occurring depression: insights from a qualitative study from the North East of England, UK.

#### Katherine L Jackson^*^, Amy Jane O'Donnell

##### Population Health Sciences Institute, Newcastle University, Newcastle upon Tyne, NE2 4AX, UK

###### **Correspondence:** Katherine L Jackson (kat.jackson@newcastle.ac.uk)

*Addiction Science & Clinical Practice* 2023, **18(Suppl 1)**: O55.

**Background:** In the UK, it is estimated that people with depression are twice as likely to engage in problematic alcohol consumption. Yet, evidence suggests that screening people with depression for heavy alcohol use is not routine in primary care or mental health services. Consequently, in this presentation we will draw on the experiences of people with co-occurring heavy alcohol use and depression to understand factors that may challenge or create opportunities for the delivery of alcohol screening and brief interventions and suggest implications for policy and practice.

**Material and methods:** Semi-structured qualitative interviews were undertaken with 40 people (22 men and 18 women) with current or recent experience of co-occurring heavy alcohol use and depression who live in the North East and Cumbria, UK. Qualitative analysis drew on interpretive description methodology.

**Results:** Three main themes were identified: (1) Lack of recognition; participants described that alcohol was sometimes not addressed or dismissed by practitioners, or that their reasons for drinking were not acknowledged in alcohol interventions (2) Nowhere to go; participants indicated that practitioners could sometimes lack knowledge or confidence in addressing alcohol use. The emphasis on themselves to manage their alcohol use and the processes of self-referral could be challenging (3) Inequities in good care; participants valued non-judgemental care from professionals who were knowledgeable about support for reducing alcohol use, but there were inconsistencies across the region.

**Conclusions:** People were receptive to the idea of discussing alcohol with practitioners. Yet, when they disclosed their alcohol use, they wanted access to appropriate advice and support as they struggle to manage their drinking without support. Screening and brief interventions by themselves will not address the needs of this population; however, they could be valuable alongside other more intense interventions which acknowledge the social context of alcohol use and depression.

### O56 A multidisciplinary approach to expanding substance use disorder treatment in an under-resourced majority Latinx community

#### Sandra J. Gonzalez^1*^, Monica Hernandez Sanchez^2^, Samuel MacMaster^1^, Roger Zoorob^1^

##### ^1^Department of Family and Community Medicine, Baylor College of Medicine, Houston, Texas, 77098, USA; ^2^Behavioral Health Solutions of South Texas, Pharr, Texas, 78577, USA

###### **Correspondence:** Sandra J. Gonzalez (sandra.gonzalez@bcm.edu)

*Addiction Science & Clinical Practice* 2023, **18(Suppl 1)**: O56

**Background:** Substance use is associated with a higher risk of HIV infection. The Rio Grande Valley (RGV) of Texas, USA, has experienced the impact of HIV/AIDS almost exclusively among young members of the Latinx community. In the RGV, it appears that the HIV epidemic is fueled by three factors: substance use, poverty, and a lack of access to services. Additionally, national cross-sectional surveys have found that racial and ethnic respondents are more likely to perceive bias and lack of cultural competence when seeking health care.

**Materials and methods:** The demonstration project was conducted by a multi-site, multi-level substance use disorder (SUD) treatment facility in south Texas in collaboration with a university partner and key stakeholders representing the HIV and recovery services community. The evaluation strategy involves a single group design, with repeated measures of program outcome indicators at program intake (baseline), program discharge, and 6-month post intake follow-up to examine changes over the course of program participation.

**Results:** A total of 307 participants were enrolled in intensive community-based outpatient SUD treatment and recovery support services over the five-year life of the project. All individuals participated in the evaluation study by completing a baseline interview and, to date, 291 have completed 6-month follow up interviews. Preliminary findings show a significant improvement in abstinence rates, a decrease in the frequency of HIV and HCV risk behaviors, increases in self-sufficiency and psychosocial functioning, and a decrease in mental health and trauma symptoms when comparing the baseline and 6-month follow up interviews.

**Conclusions:** The preliminary findings of this evaluation study suggest that a multidisciplinary, community-based approach to treatment may increase engagement and result in positive treatment outcomes among participants. The study also sheds an important light on the unique needs of Latinx people with substance use disorders and subpopulations, including sexual minority individuals.

### O57 Can single-item screening questions predict future homelessness among emergency department patients with drug or unhealthy alcohol use?

#### Kelly M. Doran^1*^, Mindy Hoang^2^, Ann Elizabeth Montgomery^3,4^, Eileen Johns^5^, Marybeth Shinn^6^, Tod Mijanovich^7^, Dennis Culhane^8^, Thomas Byrne^9^

##### ^1^Departments of Emergency Medicine and Population Health, NYU School of Medicine, New York, NY, USA; ^2^University of Cincinnati College of Medicine, Cincinnati, OH, USA; ^3^School of Public Health, University of Alabama at Birmingham, Birmingham, AL, USA; ^4^Birmingham Veterans Affairs Health Care System, Birmingham, AL, USA; ^5^NYC Center for Innovation through Data Intelligence, New York, NY, USA; ^6^Department of Human and Organizational Development, Peabody College, Vanderbilt University, Nashville, TN, USA; ^7^Department of Applied Statistics, Social Sciences, and Humanities, NYU Steinhardt School, New York, NY, USA; ^8^School of Social Policy and Practice, University of Pennsylvania, Philadelphia, PA, USA; ^9^School of Social Work, Boston University, Boston, MA, USA

###### **Correspondence:** Kelly M. Doran (kelly.doran@nyulangone.org)

*Addiction Science & Clinical Practice* 2023, **18(Suppl 1)**: O57

**Background:** Despite growing interest in screening and interventions for substance use among emergency department (ED) patients, there has been little examination into concurrent screening for patients & significant intersecting social needs such as housing insecurity. In this study we examine performance of two single-item screening questions assessing self-perceived risk of future homelessness among ED patients with drug or unhealthy alcohol use.

**Materials and methods:** We conducted a prospective cohort study of a randomly selected sample of adult patients at an urban public hospital ED. Patients completed a questionnaire that included two single-item screening questions on self-perceived risk for future housing instability and homelessness. Questionnaires were linked to city administrative data, allowing us to assess patients & subsequent shelter entry. We examined sensitivity, specificity, positive predictive value (PPV), and area under the receiver operating characteristic (AUROC) curve of each screening question in predicting shelter entry 2-, 6-, and 12-months post-ED visit.

**Results:** The final study analytic sample included 701 participants who screened positive for drug use or unhealthy alcohol use (using single-tem screeners) and were not homeless at baseline. Prevalence of shelter entry within 2-, 6-, and 12-months of the ED visit was 4.6%, 6.7%, and 10.1%, respectively. For both single-item homelessness risk screening questions, participants who answered affirmatively had significantly higher likelihood of future shelter entry at each time point. Sensitivity of the questions at various time points ranged from 0.27‚Äì0.65, specificity from 0.71‚Äì0.95, PPV from 0.10‚Äì0.40, and AUROC from 0.61‚Äì0.75.

**Conclusions:** Two single-item screening questions assessing self-perceived risk of future housing instability and homelessness among ED patients with drug or unhealthy alcohol use had adequate to good performance in predicting future shelter entry. Similar questions could be added to ED-based substance use interventions, with positive screens prompting interventions such as referral to community-based homelessness prevention services.

## Poster presentations

### P1 Evaluating barriers and facilitators to implementing adolescent screening, brief intervention, and referral to treatment education in social work and nursing curriculum using the CFIR model

#### Adrienne H. Call^1*^, Hildie Cohen^1^

##### NORC, University of Chicago, Chicago, IL 60603, USA

###### **Correspondence:** Adrienne H. Call (call-adrienne@norc.org)

*Addiction Science & Clinical Practice* 2023, **18(Suppl 1)**: P1

**Background:** Screening, Brief Intervention, Referral to Treatment (SBIRT) is an evidenced-based model to deliver prevention, early intervention, and treatment services for people with substance use disorders and those at risk of developing them. NORC, in collaboration with leading professional associations, subject matter experts, and technology partner Kognito, developed and evaluated an Adolescent SBIRT Curriculum to train the current and future workforce on screening and intervening for substance use and co-occurring mental health risks. NORC and IRETA conducted a retrospective analysis using the Consolidated Framework for Implementing Research (CFIR) model to identify barriers and facilitators to implementing the Adolescent SBIRT curriculum.

**Materials and methods:** NORC collected data from progress reports, learning collaborative calls, and implementation calls with participating nursing and social work schools. Nearly 180 individual statements were extracted from the data for analysis. Two raters independently reviewed each of the statements and categorized them according to the CFIR model. Each statement was grouped within one of the five CFIR domains and assigned a specific construct based on relevancy. Coding was later assessed for agreement.

**Results:** Barriers and facilitators related to the Inner Setting arose most often with 85 (47%) of statements meeting the domains criteria. Though less common, 39 (22%) statements were associated with Process, followed by Characteristics of Individuals, and Intervention Characteristics, each with 25 (14%) statements. Only 5 statements (3%) met the CFIR definition for Outer Setting, suggesting that such external factors impacted implementation the least.

**Conclusions:** Inner Setting, Process, and Characteristics of Individuals CFIR domains had the largest impact on curriculum implementation. The more access to materials and training, the more likely people were to feel comfortable and implement. Continual engagement and feedback opportunities between stakeholders and implementation teams is critical, as is ongoing access to the training curriculum and implementation support materials.

### P2 Brief intervention for patients using psychoactive substances

#### Angela Maria Mendes Abreu^1*^, Riany Brites^2^, Sonia Sueli S. E.Santo^1^, Larissa Mattos^1^, Marcia Cesar^1^

##### ^1^Federal University of Rio de Janeiro Health Science Center Anna Nery Nursing School Public Health Department of Nursing, Rio de Janeiro, RJ, Brazil; ^2^Federal University of Rio de Janeiro, Occupational Health Service. Rio de Janeiro, RJ, Brazil

###### **Correspondence:**Angela Maria Mendes Abreu (angelamendesabreu@gmail.com)

*Addiction Science & Clinical Practice* 2023, **18(Suppl 1)**: P2.

**Background:** About 275 million people used drugs worldwide in the last year, while more than 36 million suffered from disorders associated with the use of psychoactive substances, according to the World Drug Report 2021. Objective To analyze the effect of Brief Intervention on reduction in the consumption of psychoactive substances.

**Methods:** Longitudinal descriptive pilot study, carried out in a medium-complexity Unit with Primary Care programs, in users of psychoactive substances, in a University Hospital, Rio de Janeiro/ Brazil, applying the ASSIST. The study population was 147 patients seen between March 2019 to March 2022, the pilot study sample consisted of 22 randomly selected patients. The cutoff point for inclusion in the sample was patients from 3 consecutive consultations, leaving 18 patients in the final sample, who were undergoing Brief Intervention consultations. Descriptive statistics were used with simple frequencies, means, raw values and percentages through univariate analysis, performed in SPSS Version 21.

**Preliminary results**: Higher frequency for males 83.3%, single 46.0%, age group over 40 years 66.7%, with an average of 45.3 years, elementary schooling 44.4%, income between 1 and 4 minimum wages 50.0%. Substances used in the last three months were cocaine 66.6% and alcohol 33.3%. Time taken in the Service until the beginning of the IB consultation was from 0 to 8 weeks. The highest frequency for drug cessation was around 1 to 12 weeks (38.8%). They reduced consumption (88.8%).

**Conclusions:** You can see the effect of consultation using the Brief Intervention technique on substance use reduction and cessation. The implementation of a protocol based on Brief Intervention became a guide for all care in the Service. Study in progress, the main results will be presented at the Inebria Congress.

### P3 Exploring the impact of employee assistance programs on the reduction of absentee hours for addiction-related presenting issue

#### Ashley Peters^1*^, Jeremy Bray^1^, David Goehner^2^, Richard Lennox^3^

##### ^1^Department of Economics, UNC Greensboro, Greensboro, NC, USA; ^2^Empathia, Waukesha, Wisconsin, USA; ^3^Chestnut Global Partners, Bloomington, IL, USA

###### **Correspondence:** Ashley Peters (arpeter2@uncg.edu)

*Addiction Science & Clinical Practice* 2023, **18(Suppl 1)**: P3

**Background:** Employee Assistance Programs (EAP’s) originally grew out of occupational counseling programs in the 1940’s which aimed to respond to alcohol-impaired employees. These then grew into more formalized occupational alcoholism programs, and eventually gave way to modern EAP’s. Here, we examine the effect on absentee hours of EAP’s as a series of brief interventions for employees with addiction-related presenting issues.

**Materials and methods:** We use data from EAP provider Empathia’s Workplace Outcomes Suite dataset. We tested for statistically significant differences in outcomes between various subgroups using one and two-sample t-testing.

**Results:** Those with addiction-related presenting issues saw a reduction of 7.38 absentee hours, compared to a reduction of 4.03 absentee hours for non-addiction-related presenting issues. Those with an alcohol-related presenting issue saw a reduction of 9.08 absentee hours. Additionally, those with addiction-related presenting issues were more likely than others to indicate that their personal issues did not interfere with work, that they were happy with their lives and work, and that they were eager to start the workday.

**Conclusions:** While Employee Assistance Programs provide a benefit to most employees who utilize them, they can provide an increased benefit to those employees that struggle with addiction. When used as a series of brief interventions and/or as part of a larger treatment plan, they have the potential to decrease absenteeism among employees with addiction issues, therefore generating a return on investment to the employer as they are able to direct fewer resources toward covering late, absent, or impaired employees.

### P4 Nalmefene prescribing in UK primary care: an overview of general patterns, insights from professionals, and implications for alcohol interventions in primary care

#### Clare Sharp^1*^, Niamh Fitzgerald^1^, Linda Bauld^2^

##### ^1^Institute of Social Marketing and Health, University of Stirling, Stirling, UK; ^2^Usher Institute, University of Edinburgh, Edinburgh, UK

###### **Correspondence:** Clare Sharp (clare.sharp1@stir.ac.uk)

*Addiction Science & Clinical Practice* 2023, **18(Suppl 1)**: P4

**Introduction:** Nalmefene, first approved for use in the UK NHS in 2013, is the first pharmacotherapy to be licensed for the reduction of alcohol consumption in patients with alcohol dependence. Marketed mainly towards prescribing in primary care, the evidence supporting its efficacy and use in this setting remans contested.

**Aims:** This study aims to describe levels of and patterns in nalmefene prescribing in UK primary care, and to provide insights into factors which may have influenced uptake of the drug.

**Methods:** A mixed-methods study including a quantitative analysis of GP prescribing data (using monthly nalmefene prescribing for GP practices in England obtained from OpenPrescribing.net and patient-level data from the Clinical Practice Research Datalink (CPRD) for patients who have received nalmefene) and semi-structured interviews (n = 19) with alcohol treatment and policy professionals.

**Results:** Nalmefene prescribing in UK primary care was low, apart from a temporary increase after nalmefene was recommended by the National Institute for Health and Care Excellence (NICE) in 2014, and prescribing was poorly aligned with the drug’s licensing conditions. Whilst marketing activities were thought to have garnered some support for nalmefene, there remained substantial barriers to its use in UK primary care, including poor compatibility with current models of alcohol treatment, and a lack of skills, resources and confidence in primary care to treat alcohol dependence.

**Discussion:** The nalmefene experience, in line with some other studies, highlights the challenges of implementing alcohol interventions in primary care, and raises questions about the primary care role in addressing alcohol problems.

### P5 A randomized iterative approach to optimizing an online substance use intervention for collegiate athletes

#### David Wyrick^1*^, Cheryl Haworth Wyrick^2^

##### ^1^UNC Greensboro, Greensboro, NC, USA; ^2^Prevention Strategies, LLC, University of North Carolina, Greensboro, NC, USA

###### **Correspondence:** David Wyrick (dlwyrick@uncg.edu)

*Addiction Science & Clinical Practice* 2023, **18(Suppl 1)**: P5

**Background:** Interventions targeting alcohol use among college students show some efficacy in RCTs. Notably, most interventions do not address the unique motivations for substance use among collegiate student-athletes and the few interventions that do only address alcohol. Furthermore, the average intervention effect sizes are typically small to moderate. Our primary objective was to maximize the impact of myPlaybook, an online substance use intervention for college student-athletes, on the two most abused substances: alcohol and marijuana.

**Methods:** We evaluated intervention lessons through three sequential optimization trials, using the Multiphase Optimization Strategy framework. Each trial used a fully powered longitudinal, randomized factorial design. We recruited and randomized N = 54 (Trial 1), N = 47 (Trial 2), and N = 42 (Trial 3) schools and invited all first-year and transfer student-athletes to participate. Student-athletes completed a baseline survey, their randomly assigned intervention lessons, and immediate posttest and 30-day follow-up surveys. Across trials, N = 3,244 (48.8% female), N = 2,837 (51.9% female), and N = 2,193 (51.4% female) completed the baseline survey and at least one posttest survey. We revised lessons that did not meet the optimization criterion (d ≥ 0.3) for the proximal outcomes.

**Results:** Trial 1: The alcohol lesson significantly improved descriptive and approval norms, and positive expectancies and the marijuana lesson significantly improved negative expectancies, but all d < 0.15. We then revised lessons to target proximal outcomes, rather than specific substances. Trial 2: the norms and expectancies lessons had some significant effects, but some d < 0.3, so all lessons were revised. Trial 3: The norms lesson improved all proximal outcomes (all d > 0.35). The expectancies lesson improved alcohol positive expectancies (d = 0.3) and marijuana negative expectancies (d = 0.16). The other lessons had no significant effects.

**Conclusions:** After three optimization trials, myPlaybook lessons had substantially stronger effects on proximal outcomes, increasing the likelihood that the intervention “package” will have a meaningful clinical impact on college student-athletes’ substance use.

### P6 Curricular innovation related to management of patients with opioid use disorder

#### Deborah S. Finnell^1*^, Tammy M. Slater^1^

##### ^1^School of Nursing, Johns Hopkins University, Baltimore, MD 21205, USA

###### **Correspondence:** Deborah S. Finnell (dfinnell@jhu.edu)

*Addiction Science & Clinical Practice* 2023, **18(Suppl 1)**: P6

**Background:** Buprenorphine prescribing for treatment of persons with opioid use disorder is heavily regulated in the U.S. Since 2016, nurse practitioners (NPs) have the legal right to provide buprenorphine treatment, albeit with completion of federally-approved education, possession of a federal waiver to prescribe buprenorphine, and within the context of their state’s scope of practice. The demand for opioid-related treatment outpaces the number of qualified providers. Adding specialized content to nursing curricula will prepare NP graduates to manage patients with opioid use disorder, including treatment with buprenorphine.

**Materials and methods:** With funding from the Substance Abuse and Mental Health Administration, the project team (NP faculty) at a large mid-Atlantic School of Nursing conducted a gap analysis of the current NP curriculum. While content related to screening, brief intervention and referral to treatment for alcohol and other drug use was included in the curriculum, opioid-specific content was lacking. The project team developed interactive learning modules to enhance opioid-related knowledge and skills for managing patients with opioid use disorder. To ensure that the content was delivered within the context and framework of existing curricula, the modules were placed in relevant courses.

**Results:** Three interactive case-based learning modules were developed and integrated into courses for NP students: Overview of Pain and Opioid Use, Addressing Stigma in Healthcare, Caring for Patients with Chronic Pain Over Time. A final module was developed to direct students to the 24-h waiver training requirements.

**Conclusions:** The case-based modules provided students with the opportunity to visualize patients with opioid use disorder at a time when the pandemic precluded clinical practice opportunities. The enhanced curriculum is the first step toward expanding the NP workforce prepared to manage patients with opioid use disorder. This sustainable curriculum holds promise for increasing the number of NPs who can prescribe buprenorphine.

### P7 Changes in alcohol use during the COVID-19 pandemic: survey among women in primary health care

#### Divane de Vargas^1*^, Erika Gisseth León Ramírez^1^, Caroline Figueira Pereira^1^, Rosa Maria Jacinto Volpato^1^, Ana Vitoria Correa Lima^1^, Jose Adelmo Da Silva Filho^1^

##### Psychiatric Nursing Department, School of Nursing University of São Paulo, São Paulo, Brazil

###### **Correspondence:**  Divane de Vargas (vargas@usp.br)

*Addiction Science & Clinical Practice* 2023, **18(Suppl 1)**: P7

**Background:** In the last decades, the women's role in the society had been changed, women began to take on multiple tasks, and face the challenges of a world built for men. During the pandemic period, this situation became worse, generating overload and stress feelings. Consequently studies warning about the change in alcohol use profile in women during the pandemic. In this way, this study aims to investigate the changes in women's pattern of alcohol use during the first twelve months of the COVID-19 pandemic and the correlated factors, in primary care health services in Brazil.

**Methods:** Cross-sectional study with a convenience sample and telephone-based interviews to identify alcohol use patterns among 3252 women from primary health care (PHC) patients during the COVID-19 pandemic. The Alcohol Use Disorders Identification Test (AUDIT-C) were used to assess the alcohol use patterns. To assess the change of alcohol use patterns, the pandemic periods were classified according to the intensity of the restriction measures during the twelve months of pandemic in three categories (Maximum restriction, lessening of restrictions and transition to eliminate restrictions).

**Results:** The mean score of AUDIT C was 2.08 (SD 2.75), indicating a trend of alcohol use among low risk and moderate risk. When analyzed the change of alcohol use by periods, the higher AUDIT C mean observed was 3.35 (SD3.04) indicating moderate risk during the transition to eliminate restrictions. On the other hand the lower mean (0.78 SD 1.71) was observed in the Maximum restriction period. Among the factors significantly related (p < 0.05) with these changes were marital status, income, education level and age.

**Conclusions:** The results of this study could be important in identifying ways of responding to the consequences of the pandemic on women's mental health and providing support for the development of strategies for the assessment and prevention.

### P8 I thought cancer was a tobacco issue: perspectives of Veterans with and without HIV on cancer risks associated with alcohol and tobacco use

#### Elsa S. Briggs^1,2*^, Madeline C. Frost^1,2^, Rachel M. Thomas^1^, Olivia V. Fletcher^1^, Kristina A. Crothers^1,3^, Clementine K. Chalal^1^, Jennifer B. McClure^4^, Sheryl L. Catz^5^, Emily C. Williams^1,2^

##### ^1^Health Services Research and Development, Center of Innovation for Veteran-Centered and Value-Driven Care, Veterans Affairs Puget Sound Health Care System, Seattle, Washington, 98108, USA; ^2^Department of Health Systems & Population Health, University of Washington School of Public Health, Seattle, Washington, 98195, USA; ^3^Division of Pulmonary, Critical Care, and Sleep Medicine, University of Washington School of Medicine, Seattle, Washington, 98195, USA; ^4^Kaiser Permanente Washington Health Research Institute, Seattle, Washington, 98101, USA; ^5^Betty Irene Moore School of Nursing, University of California, Davis, Sacramento, California, 95817, USA

###### **Correspondence:** Elsa S. Briggs (elsa8@uw.edu)

*Addiction Science & Clinical Practice* 2023, **18(Suppl 1)**: P8

**Background:** Feedback linking substance use to health is a key component of brief intervention. Both U.S. Veterans and people living with HIV (PWH) experience higher rates of unhealthy alcohol and tobacco use than non-Veterans and people without HIV (PWoH) and are often more susceptible to adverse health outcomes associated with these substances, including many cancers. Understanding awareness of cancer risk related to alcohol and tobacco use and their co-occurrence could inform brief interventions for both substances.

**Materials and methods:** We conducted semi-structured interviews exploring awareness of and beliefs about how alcohol and tobacco use, alone and together impact cancer risk in a diverse sample of Veterans Health Administration (VA) patients with and without HIV. Participants were identified from national electronic health record data in VA, using a stratified purposive sampling frame to ensure a range of alcohol and tobacco use and diverse lived experiences and identities. Interviews were conducted via telephone, recorded, and transcribed and analyzed using a Rapid Assessment Process.

**Results:** Among 41 participants (46% PWH, 54% PWoH; 73% male, 39% black), 63% reported current smoking and most screened positive for unhealthy alcohol use (mean AUDIT-C score = 5.9). For patients with and without HIV, preliminary analyses showed awareness of tobacco-related cancer risk was high, while awareness of alcohol-related cancer risk was very limited, with no notable differences between groups. Despite limited awareness, some participants felt it was plausible that cancer risk would increase for individuals with co-occurring alcohol and tobacco use.

**Conclusions:** Among PLWH and PWoH, there was minimal awareness of how alcohol or co-occurring alcohol and tobacco use impact long-term cancer risk. Findings suggest that, among PWH and PWoH, there is a need for increased and improved messaging around cancer risk related to alcohol and co-occurring alcohol and tobacco use. These findings could inform future iterations of brief interventions for both substances across populations.

### P9 Acceptability of a brief personalized prevention model for reducing alcohol and other substance use risk among youth: findings from a qualitative interview study

#### Elissa R. Weitzman^1,2,3*^, Laura M Blakemore^1^, Joe Kossowsky^3,4^, Sharon Levy^3,5^

##### ^1^Division of Adolescent/Young Adult Medicine, Boston Children's Hospital, Boston, MA 02115; ^2^Computational Health Informatics Program, Boston Children's Hospital, Boston, MA 02115, USA; ^3^Department of Pediatrics, Harvard Medical School, 25 Shattuck Street, Boston, MA 02115, USA; ^4^Department of Anesthesiology, Critical Care and Pain Medicine, Boston Children's Hospital, Boston, MA 02115, USA; ^5^Adolescent Substance Use and Addiction Program, Division of Developmental Medicine, Boston Children's Hospital, Boston, MA 02115, USA

###### **Correspondence:** Elissa R. Weitzman (elissa.weitzman@childrens.harvard.edu)

*Addiction Science & Clinical Practice* 2023, **18(Suppl 1)**: P9

**Background:** Humans are compelled by notions of biological vulnerability to disease, driving direct-to-consumer genetic testing. One in 25 US adults obtained personalized genetic test reports in 2017, and substance use disorder is an area of peak interest including among adolescents who stand to benefit greatly from early preventive interventions. Still, little is known about the acceptability of a personalized brief intervention involving return of personal genetic risk information to adolescents to motivate alcohol or other substance use risk limiting behavior.

**Objective:** To explore acceptability of a model for returning individual genetic testing information to adolescents to motive risk reducing behaviors.

**Methods:** Trained research staff virtually interviewed adolescents and adults recruited from clinical and community settings using a semi-structured guide about thoughts and concerns regarding genetic testing and personalized prevention, perceived risks, and benefits. Qualitative interviews were transcribed, coded, and thematically analyzed.

**Results:** Participants (N = 9) ranged in age from 16 to 63 (Ave.26.7), of which, 67% identified as female and 22% had previously received genetic testing. Overall, attitudes were strongly favorable toward the model. Perceived benefits included informing decisions to modify behavior early in life to avoid future risk, opportunity to share important health information with family members for their protection, confirmation of and insight into health problems. Potential harms included the potential for experiencing anxiety, increasing risk behaviors on the false assumption of protection should a genetic test be negative, stigma. Most participants favored integration of a personalized prevention model into routine pediatric care with parental involvement for adolescents.

**Conclusions:** High interest in and acceptability of a personalized prevention model were found along with appreciation for how understanding genetic risk for alcohol and other substance use disorders might motivate health decision making and risk modifying behaviors. Further research is needed to elucidate operational, ethical, and communications strategies to advance this model.

### P10 Implementation and impact of adolescent SBIRT training for substance use and co-occurring mental health risks

#### Hildie Cohen^1*^, Tracy L. McPherson^1^, Giana Calabrese^1^

##### ^1^Public Health, NORC at the University of Chicago, Chicago, IL 60603, USA

###### **Correspondence:** Hildie Cohen (cohen-hildie@norc.org)

*Addiction Science & Clinical Practice* 2023, **18(Suppl 1)**: P10

**Background:** Screening, Brief Intervention, Referral to Treatment (SBIRT) is an evidenced-based model to deliver prevention, early intervention, and treatment services for people with substance use disorders and those at risk of developing them. NORC, in collaboration with leading professional associations, subject matter experts, and technology partner Kognito, developed and evaluated an Adolescent SBIRT Curriculum to train the current and future workforce on screening and intervening for substance use and co-occurring mental health risks such as suicide risk. Since 2015, over 600 academic institutions and organizations have implemented the curriculum and 25,000 individuals have been trained via virtual and classroom instruction. This presentation will discuss the Adolescent SBIRT Curriculum resources and its large-scale implementation including the results of an evaluation of the virtual SBIRT-Suicide Prevention Training.

**Materials and methods:** The Adolescent SBIRT Initiative at NORC engaged a national learning collaborative and steering committee to develop, implement, and evaluate the curriculum. In addition, a virtual SBIRT-Suicide Prevention Training with dedicated website with resources were created.

Over 3,550 individuals attended the virtual SBIRT-Suicide Prevention Training. A posttest and 2-month follow up survey was administered to assess learner outcomes and practice uptake with a sample of 331 attendees completing both the posttest and 2-month posttest.

**Results:** Findings indicated 64% of trainees reported sharing information with colleagues, 20% trained other health professionals on utilizing adolescent screening tools for suicide risk; and 17% implemented screening and/or brief interventions to assess suicide risk. Attendees showed a statistically significant increase in confidence in screening for alcohol and drug use and suicide risk using a validated tool at 2-months post webinar.

**Conclusions:** Findings suggest that large-scale implementation of the Adolescent SBIRT Curriculum focusing on substance use and suicide prevention is feasible and can positively build workforce capacity to deliver key components of SBIRT in a short period of time.

### P11 Interventions targeting women at risk of drinking alcohol during pregnancy within the FAR SEAS pilot project

#### Katarzyna Okulicz-Kozaryn^1*^, Carla Bruguera Soler^2^, Lidia Segura Garcia^2^, Joan Colom Farran^2^, Fleur Braddick^3^, Emanuele Scafato^4^, Claudia Gandin^4^, Alice Matone^4^, Marta Zin-Sędek^5^

##### ^1^Children and Adolescent Health Department, Institute of Mother and Child, Warsaw, 01-211, Poland; ^2^ASPCAT, GENCAT Roc Boronat 81-95, 08005 Barcelona, Spain; ^3^Clínic Addictions Research Group (GRAC), Hospital Clínic, Barcelona, 08036, Spain; 4National Observatory on Alcohol, National Centre on Addictions and Doping, Istituto Superiore di Sanità, Rome, 00161, Italy; ^5^Research, Monitoring and International Cooperation Department, National Centre for Prevention of Addictions (KCPU), Warsaw, Poland

###### **Correspondence:** Katarzyna Okulicz-Kozaryn (katarzyna.okulicz@imid.med.pl)

*Addiction Science & Clinical Practice* 2023, **18(Suppl 1)**: P11

**Background:** FAR SEAS is a tendered service contract for the European Commission under the EU Health Programme, aimed at reducing alcohol exposed pregnancies (AEP) to improve public health. One FAR SEAS task is to pilot the implementation of good practice interventions in the Mazovian region (Poland).

**Methods:** Staff (n = 30) from local-level services in 4 towns (social, therapeutic and psychological), screened women of child-bearing age (pregnant and not pregnant) for alcohol risky use (using AUDIT-C) and psychosocial risks. Interventions ranged from simple feedback, via brief intervention (BI), motivational interviewing (MI) sessions, to referral to other specialist and individualized care package, and were tailored to the needs of the women according to their level and type of risk and their reproductive status. 441 women of child-bearing age, including 42 pregnant women (9,5%), were recruited to the pilot study (in low, moderate, and high-risk groups: 70%, 23%, 7%, respectively).

**Results:** At least one intervention was offered to 95% of screened women. The number of interventions increased with the risk level ‚Äì at least two types of interventions were received by 7% of women at low risk, 75% moderate risk and 84% high risk of AEP. Three or more types of interventions were received by 1%; 15% and 36% of participants at the respective the risk levels. The professionals‚Äô first-choice intervention was typically feedback and/or BI, while, for those receiving multiple interventions, MI and referrals to specialists were more common.

**Conclusions:** Considering that most project staff only became acquainted with the screening, BI and MI procedures during the FAR SEAS training, the implementation rates seem satisfactory. However, the success of the project will be determined only by the final evaluation data (due end of June this year), which will allow us to assess the impact of the interventions on women's knowledge and behavioral outcomes.

### P12 Transformation: integrating SBIRT practice within US accredited addiction medicine MD fellowship program

#### Laura J. Veach^1*^, Jie Cao^2^

##### ^1^Department of Surgery-Trauma, Wake Forest University School of Medicine, Winston Salem, NC 27157, USA; ^2^MBBS, MS, MD, Wake Forest University School of Medicine, Winston Salem, NC 27157, USA

###### **Correspondence:** Laura J. Veach (lveach@wakehealth.edu)

*Addiction Science & Clinical Practice* 2023, **18(Suppl 1)**: P12

**Background:** Efforts to set quality U.S. addiction medicine training standards were first implemented by the American College of Academic Addiction Medicine with 86 programs now listed. In 2018, the prestigious American College of Graduate Medical Education (ACGME) began historic accreditation of addiction medicine programs. The ACGME fellowship involves a multispeciality training focused on those impacted by substance use disorders (SUD), unhealthy substance use, and other addiction-related disorders. Important areas of prevention, clinical assessment, various types of SUD treatments, and ongoing care are required. While didactic standards include teaching elements of screening, brief intervention, and referral to treatment (SBIRT) practices, few models involve integration of SBIRT practices throughout the fellowship program. Previous criticisms of large scale U.S. SBIRT implementation found that very few physicians continued SBIRT practices.

**Methods:** As a Clinical Surgery Professor at Wake Forest University School of Medicine, core faculty member of our ACGME-accredited program, and integral in the planning in 2017 of this new 2-year fellowship program, innovative integration of addiction medicine didactics with pioneering SBIRT practices has been at the forefront. As the Founding Director of the Addiction Research & Clinical Health M.S. program, integrating the fellows on a continual basis with graduate learners practicing SBIRT at the hospital bedside underscored important immersive education.

**Results:** Data related to immersive SBIRT instructional methods since 2018 will be shared. Findings of post-fellowship SBIRT practices in medical settings and the practice of addiction medicine as their primary specialty utilizing SBIRT practices will be reviewed. This presentation outlines immersive pedagogy using SBIRT in ongoing medical practice and its implications for future research.

**Conclusion**: Ongoing examination of key contributors to SBIRT implementation suggests immersive medical education is an area for further research. Substantial effort in this research may involve a continued emphasis on addiction medicine integrated with SBIRT practices.

### P13 Implementation of systematic screening, brief intervention, and management of alcohol use disorders: an adaptation of the SPARC trial intervention for small primary care clinics

#### Leah K. Hamilton^1*^, Mariah Black-Watson^2^, Anya Day^2^, Christine Stanik^2^, Rachelle May-Maki^2^, Katharine Bradley^1^

##### ^1^Kaiser Permanente Washington Health Research Institute, Seattle, WA, 98101; ^2^Altarum Institute, Ann Arbor, MI 48105, USA

###### **Correspondence:** Leah K. Hamilton (leah.k.hamilton@kp.org)

*Addiction Science & Clinical Practice* 2023, **18(Suppl 1)**: P13

**Background:** The Sustained Patient-centered Alcohol-Related Care (SPARC) implementation trial used three implementation strategies; practice facilitation, EHR clinical decision support, and performance feedback to improve alcohol-related primary care (PC) along with other behavioral health integration. However, it is unknown whether this approach to implementing alcohol-related care could be adapted to small, independent PC practices. Michigan SPARC tested an adaptation of the SPARC implementation intervention in small PC practices in Michigan and Indiana (March 2020-Dec. 2022). Results to date are presented.

**Materials and methods:** 28 practices were recruited. Clinics were assigned a practice facilitator who provided Continuing Medical Education training and guided alcohol-related implementation for 6 months using SPARC strategies; practices were also provided a patient decision aid to support shared decision making about AUD. Data were collected by manual chart review (n = 7) or electronic EHR data (n = 2). Analyses were at the practice level.

**Results:** 14 practices completed CME and engaged with practice facilitators; 13 practices integrated paper screening using the AUDIT-C into their workflow. No practice was able to build prompts for screening into their EHR, and only nine practices (so far) obtained baseline EHR data for performance feedback. At baseline (n = 9), mean prevalence of alcohol screening was 26% (0–100%) of patients, with mean practice prevalence of positive screens 0–54.5%. Mean baseline prevalence of documented AUD diagnosis was 0.07% (range: 0.09–0.3%). Five practices that collected 6-month follow-up data (so far) had a mean screening prevalence of 25% (6–45%) of patients, with a mean practice prevalence of positive screens 16% (0–42%), and AUD diagnoses 0.7% (0%-2%).

**Conclusion**: Michigan SPARC was heavily impacted by both COVID and non-COVID-related barriers, resulting in uneven implementation and the need for iterative adaptations to the SPARC approach. To date, the implementation has not increased prevalence of screening overall and minimally increased AUD diagnosis.

### P14 Gaps between teaching and implementing BI in public services in Brazil

#### Liz Paola Domingues^1*^, Danilo Polverini Locatelli^2^, André Bedendo^3^, Ana Regina Noto^1^

##### ^1^Department of Psychobiology, Federal University of São Paulo, São Paulo, Brazil; ^2^Research Incentive Fund Association (AFIP), São Paulo, Brazil; ^3^Department of Health Sciences, University of York, York, UK

###### **Correspondence:** Liz Paola Domingues (liz.paola@unifesp.br)

*Addiction Science & Clinical Practice* 2023, **18(Suppl 1)**: P14

**Background:** Despite promising results of Brief Intervention (BI) in reducing alcohol and other drugs use, its implementation in professional routine is very challenging. Studies on training program outcomes show that professionals struggle to implement the content in their routine practice.

**Objective:** This study has aimed to assess the implementation challenges of BI in the work routine following a multi-professional training on alcohol and drugs in São Paulo—Brazil.

**Methodology:** Between 2016 and 2017, a Regional Reference Center (CRR) located at Universidade Federal de São Paulo (Brazil) offered 40 h trainings to professionals working in public institutions of health, education, social assistance, justice, and the safety system; addressing topics from basic knowledge of drug addiction to the development of specific skills, such as screening and BI. A year later, 28 of the 310 professionals who concluded the training were assessed at follow up interviews. They were randomly selected and interviewed using semi-structured script. Data were collected until reaching theoretical saturation. Two collective interviews were also conducted with 09 experts/professors who offered the referred trainings.

**Results:** The data indicated that after a year, trained professionals from different working areas recognized the importance of the new practices, but implementation was challenging. The professionals reported the lack of leaders’ support as a major barrier for implementation of screening and BI, followed by lack of time due to work overload. The experts reinforced the need to teach the use of protocols consistent with the professionals’ real routine. They also indicated that more traditional assessment strategies may not access real changes related to training.

**Conclusion:** This study shows that the implementation of BI and other practices is difficult to achieve unless stakeholders and leaders are also appreciative and supportive of the implementation process. It is also important that the protocols reflect the professional’s routine.

### P15 Early detection and brief intervention for sexual health in addiction settings

#### Marianne Hochet^1*^, Nicolas Bonnet^1^

##### ^1^RESPADD (French Network for addiction prevention), Paris, 75014, France

###### **Correspondence:** Marianne Hochet (marianne.hochet@respadd.org)

*Addiction Science & Clinical Practice* 2023, **18(Suppl 1)**: P15

**Background:** There are limits both from primary care health professionals and from patients to discuss sexual health issues. The first one might feel uncomfortable with this topic and in need of more information and resources. Patients could be embarrassed or shameful to talk about it. In the meantime, people are more likely to meet a primary care health professional like their general practitioner and they mainly consider that their doctor should ask them about their sexual health. Moreover, sexual health troubles are often linked with an addictive behaviour like drug consumption. Thus, the French addiction prevention network, RESPADD, worked on a practical guidebook to support primary care health professionals while talking of sexual health in addiction settings.

**Materials and methods:** In order to write this practical tool, several methods were used like a national and international literature review. We also contacted international organisations to know more about existing data and resources. We then gathered evidence-based questionnaires which can be used by healthcare professionals. All the collected data were discussed with a multi-professional working group, gathering governmental bodies, associations, healthcare professionals from both sexual health and addiction fields. The group met several times to think about the guidebook, its content, the format and the best way to conduct an early detection and brief intervention for sexual health in addiction settings.

**Results:** We have set up and regrouped a working group several times and the main results are: first a real need identified for the guidebook but also to talk and think about this theme. And finally, the publication of the guidebook distributed electronically and in printed form in 2000 copies.

**Conclusions:** The guidebook has been well accepted by health professionals and is often ordered. It answers to a real need and must be completed by trainings.

### P16 Worldwide distribution of studies on brief intervention for alcohol and its relationship with countries’ alcohol dependence rates

#### Micaella Leandro Silva^1*^, Wellington Francisco Rodrigues^2^, Maria Lucia Oliveira Souza Formigoni^3^

##### ^1^Wellington Francisco Rodrigues, Psychobiology, University of São Paulo, São Paulo, Brazil; ^2^Health Sciences, Federal University of Triangulo Mineiro, Uberaba, Brazil; ^3^Departamento de Psicobiologia, Escola Paulista de Medicina, Universidade Federal de São Paulo, São Paulo, Brazil

###### **Correspondence:** Micaella Leandro Silva (micaella.leandro@unifesp.br)

*Addiction Science & Clinical Practice* 2023, **18(Suppl 1)**: P16

**Background:** Studies on Brief intervention developed in different countries indicated its effectiveness in reducing harmful alcohol consumption.

**Objectives:** To describe the association between the frequency and types of studies on brief intervention to reduce alcohol use with the prevalence of alcohol dependence in the countries where the studies were developed.

**Methods:** We carried out a systematic review using the mesh terms “crisis intervention” and “alcohol drinking”, as well as their respective entry terms for the selection of studies (Boolean operators "or" and "and") over a period of 10 years (2012 to 2021), following the recommendations of Prisma (2020). Medline/ Pubmed, Cochrane and Prospero (grey literature) databases were consulted, without language restriction. We extracted the variables "authors", "year of study", "type of study" and "country" to evaluate their association with the number and kind of studies. The frequencies of adults (15 + years) suffering from disorders attributable to alcohol consumption (ICD-10: F10.1 and F10.2) for different countries and continents (year 2018 data—last update) were obtained in the Global Health Observatory (World Health Organization).

**Results:** In the period evaluated, most of the studies were conducted in the US (48.7%), followed by the UK and Australia (9.2% each). The American continent was responsible for 56.6% of the studies, followed by the European continent with 26.3%. All other countries were responsible for 17.1% of the studies. Considering the regional prevalence of alcohol dependence, the Americas had the highest prevalence (4.1%), followed by Europe (3.7%). The number of studies was positively correlated with alcohol dependence rates (Spearman’s rho = 0.82).

**Conclusion:** We found a correlation between the number of studies on brief intervention for reducing alcohol and the alcohol dependence rates of the countries where they were performed.

### P17 Comfort and beliefs around SBIRT and sexual risk screening among U.S. trauma surgery providers

#### Michael S. Argenyi^1^, William McGill^2^, Laura J. Veach^3^, Preston R. Miller III^3^

##### ^1^Department of Anesthesiology, Wake Forest University School of Medicine Winston-Salem, NC 27157, USA; ^2^Monalco Research, Port Washington, WI, USA; ^3^Department of General Surgery, Wake Forest School of Medicine, Winston-Salem, NC 27157, USA 

###### **Correspondence:** Michael S. Argenyi (drargenyi@gmail.com)

*Addiction Science & Clinical Practice* 2023, **18(Suppl 1)**: P17

**Background:** Since 2006, U.S. Surgical Specialty Trauma Centers are mandated to implement SBIRT services for alcohol-related injuries and complications. However, since this key care initiative impacts: surgeons, advanced practice providers, fellows, and residents, particularly surrounding incorporation of sexual behavior such as sexualised drug use. This presentation will review quantitative and qualitative findings of an institutional IRB-approved study exploring SBIRT and sexual health beliefs and comfort among trauma physicians and trainees before and after an educational didactic on SBIRT and brief advice.

**Materials and methods:** Participants received a pre-training survey, 1-h didactic, and post-training didactic. Participants self-assessed comfort levels with SBIRT implementation, substance use-related sexual behaviors, and addiction care pre- and post-training. Responses were analyzed using paired descriptive statistics. A subset participated in qualitative interviews. Transcripts were coded and analyzed for emerging themes.

**Results:** Participants pre- and post-training survey results will be presented. The qualitative interview analyses will focus on emerging themes regarding implementing SBIRT and brief advice, given a case study of a trauma patient with problematic sexualized drug use. Both qualitative and quantitative analyses will demonstrate whether the didactic was effective in changing comfort levels and beliefs about SBIRT, brief advice, and sexualized drug use.

**Conclusions:** Improved training is impactful and provider comfort in implementing brief advice and support SBIRT policy for the trauma patient, particularly in the setting of sexualized drug use, are key areas to address. A future multisite trial is recommended, alongside patient chart data analysis, to objectively determine the observed rates of provider advice or referral. Examination of survey items post didactic can yield beneficial indicators for future study in other U.S. trauma care institutions.

### P18 Lack of demographic differences in effectiveness of self-administered screening and follow-up treatment for mental health and substance use in HIV primary care

#### Michael. J. Silverberg^1*^, Tory Levine-Hall^1^, Varada Sarovar^1^, Alexandra N. Lea^1^, Amy S. Leibowitz^1^, Michael A. Horberg^2^, C. Bradley Hare^3^, Mitchell N. Lu^4^, Jason A. Flamm^5^, Derek D. Satre^1,6^

##### ^1^Division of Research, Kaiser Permanente Northern California, Oakland, CA, USA; ^2^Mid-Atlantic Permanente Research Institute, Kaiser Permanente Mid-Atlantic States, Rockville, MD, USA; ^3^San Francisco Medical Center, Kaiser Permanente Northern California, San Francisco, CA, USA; ^4^Oakland Medical Center, Kaiser Permanente Northern California, Oakland, CA, USA; ^5^Sacramento Medical Center, Kaiser Permanente Northern California, Oakland, CA, USA; ^6^Department of Psychiatry and Behavioral Sciences, University of California, San Francisco, CA, USA

###### **Correspondence:** Michael. J. Silverberg (michael.j.silverberg@kp.org)

*Addiction Science & Clinical Practice* 2023, **18(Suppl 1)**: P18.

**Background:** Substance use (SU), depression and anxiety are common among persons with HIV (PWH) yet often go unrecognized and untreated. We evaluated the effectiveness of computerized SU and mental health screening and behavioral treatment among PWH.

**Materials and methods:** The Promoting Access to Care Engagement (PACE) trial enrolled 2,865 PWH from 2018–2020 in 3 HIV primary care clinics in Kaiser Permanente Northern California. PWH received bi-annual questionnaires consisting of the Tobacco, Alcohol, Prescription medication, and other Substance use (TAPS) instrument, Patient Health Questionnaire-9 (PHQ-9), and Generalized Anxiety Disorder-2 (GAD-2). Results were integrated into electronic health records and were visible to physicians and behavioral health specialists (BHS) for clinical follow-up. We measured changes (between consecutive screens) in SU (score ranges from 0 [no risk] to 3 [high risk] or 4 ([for alcohol only]), PHQ-9 (range 0 to 27), and GAD-2 (range 0 to 6) among PWH who screened positive for SU, depression (PHQ-9 ‚â•10) or anxiety (GAD-2 ‚â•3). Changes were evaluated with generalized linear models, with adjustment for demographics, HIV risk, CD4, HIV RNA, SU, depression/anxiety, and BHS visits.

**Results:** Of 2,865 PWH screened, 403 screened positive and had a 6-month follow-up screen (61% •50 years; 51% White; 77% men who have sex with men). 32% screened positive for tobacco, 36% for alcohol, 27% for cannabis, 27% for depression and 31% for anxiety. We noted decreases (P < 0.05) for tobacco, alcohol, cannabis, PHQ-9, and GAD-2 over time. Decreases were similar (P > 0.05) by gender, age and race/ethnicity for most outcomes, except for greater decreases in alcohol for ages 50–59 vs. 18–49 years (difference -0.49; p = 0.004) and greater decreases in GAD-2 for black compared with white PWH (difference -0.89; p = 0.049).

**Conclusions:** Routine SU and mental health screening and treatment in HIV primary care has potential to improve outcomes broadly by age, gender and race/ethnicity.

### P19 Project lifeline pilot: implementing SBIRT in rural community pharmacies to address opioid overdoses and substance use disorder

#### Renee M. Cloutier^1*^, Abigail Talbert^1^, Joseph Weidman^2^, Janice L. Pringle^3^

##### ^1^Program Evaluation and Research Unit (PERU), University of Pittsburgh School of Pharmacy, Pittsburgh, PA 15206, USA; ^2^Janssen Pharmaceuticals, A Johnson and Johnson Company, West Chester, PA 19380, USA; ^3^Program Evaluation and Research Unit (PERU), University of Pittsburgh School of Pharmacy, Pittsburgh, PA 15206, USA

###### **Correspondence:** Renee M. Cloutier (renee.cloutier@pitt.edu)

*Addiction Science & Clinical Practice* 2023, **18(Suppl 1)**: P19

**Background:** There is emerging recognition of the benefits of implementing screening, brief intervention, referral to treatment (SBIRT) in pharmacy settings as they have more accessible locations and hours than other healthcare structures. Pharmacists already monitor prescriptions for contraindications, provide patient and provider education, and are the final scheduled face-to-face interaction with patients at their highest risk moments (i.e., filling prescriptions). Project Lifeline is a public health initiative to provide training and technical support to community pharmacies implementing SBIRT for substance use disorder (SUD) and providing harm reduction services.

**Materials and methods:** Between 2018–2020, eight community pharmacies were recruited from rural counties in Pennsylvania, USA. Patients receiving/dropping off a Schedule II prescription were invited to engage in SBIRT and offered naloxone. Descriptive statistics on patient screening summarized the patients served. Key informant interviews with pharmacy staff were analyzed to categorize barriers and facilitators to SBIRT implementation.

**Results:** 3,514 screens were completed for 3,122 unique adult patients (54.1% male, 45.9% female; < 0.01% nonbinary; 96.4% White; 3.6% non-White); 2.2% were indicated for a BI, and 1% were indicated for SUD RT. Of indicated patients, 44.2% accepted BI and 13.8% RT. 372 patients received naloxone. Facilitators identified via key informant interviews highlighted the importance of person-centered staff education, role-playing, anti-stigma training, and integrating activities into patient-care workflows. Barriers included lack of SBIRT service reimbursement as well as addiction stigmas held by staff members and patients.

**Conclusions:** Results demonstrate the feasibility of implementing SBIRT in community pharmacies, including the potential to improve identifying patients at risk for overdose, provide real time brief interventions/harm reduction tools, and refer to local services. Findings currently serve as the foundation for an ongoing project aimed at demonstrating the impact of SBIRT implementation on down-stream health care costs in additional counties which will also be discussed.

### P20 Evaluating training in alcohol screening and brief intervention from the participants perspective

#### Tadeja Hočevar^1*^, Karmen Henigsman^1^, Pika Založnik^1^, Marko Kolšek^2^

##### ^1^National Institute of Public Health, Slovenia; ^2^Department of family medicine, Medical faculty, University of Ljubljana, Slovenia

###### **Correspondence:** Tadeja Hočevar (Tadeja.Hocevar@nijz.si)

*Addiction Science & Clinical Practice* 2023, **18(Suppl 1)**: P20

**Background:** Until 2022 Alcohol screening and brief intervention (SBI) in Slovenia has been practiced systematically by general practitioners. There was a need to renew the training programme and to broaden the SBI practice to other profiles. Within the national project, we formed a more in depth training modules, trained the selected profiles and piloted the SBI practice in 18 areas across the country.

One of the objectives of our research was to learn about participants’ opinions regarding contents of the SBI training.

**Materials and methods:** Phase one, before piloting the approach: A cross sectional survey was conducted before and after a 32-h long training. A total of 263 participants, primary health care providers and social workers participated. Participants filled in surveys regarding the content of the training, in 5 sections: self-assessment of knowledge on alcohol related issues, of use of motivational interviewing elements, and of their effectiveness in using SBI, opinions regarding the legitimacy of discussing alcohol drinking, and evaluation of responses to two scenarios. They also addressed their further needs for an effective use of SBI.

Phase two, during piloting the SBI practice: We conducted training in form of monthly group sessions with participants/piloting experts and offered them individual support by phone/email. On three occasions we asked them about the usefulness of them both. We used the same method as in phase one.

**Results:** Phase one: In almost all sections the data showed statistically significant improvement after the training. Participants emphasized the need for more practice in order to achieve greater effectiveness.

Phase two: Participants found training motivational and useful. There was a non-response bias.

**Conclusions:** According to the results of our research, the training was successful, engaging in some form of training for doing SBI should be a continuous part of practicing the measure.

### P21 Getting fit for change: exercise as treatment for alcohol use disorder

#### Victoria Gunillasdotter*, Sven Andréasson, Maria Jirwe, Örjan Ekblom, Mats Hallgren

##### ^1^Karolinska Institutet, Department of Global Public Health, Substance use and Social Environment, Stockholm, Sweden; ^2^Department of Public Health Sciences, Karolinska Institutet, Stockholm, Sweden; ^3^Centre for Psychiatry Research, Department of Clinical Neuroscience, Karolinska Institutet & Stockholm Health Services, Stockholm, Sweden; ^4^Department of Neurobiology, Care Sciences and Society, Karolinska Institutet, Huddinge, Sweden; ^5^Department of Health Sciences, the Swedish Red Cross University College, Huddinge, Sweden; ^6^Swedish School of Sport and Health Science, Stockholm, Sweden

###### **Correspondence:** Victoria Gunillasdotter (victoria.gunillasdotter@ki.se)

*Addiction Science & Clinical Practice* 2023, **18(Suppl 1)**: P21

**Background:** Alcohol use disorders (AUD) are highly undertreated. Major barriers to treatment are stigma and the desire to self-manage the problem. In addition to health risks of heavy drinking, hazardous drinkers are reported to be less physical active than non-hazardous drinkers. Exercise is a non-stigmatizing treatment option with synergistic effects on physical fitness, somatic health, and mood. Prior research has demonstrated the potential role of exercise as treatment for AUD, but robust studies in non-treatment seeking individuals are lacking.

**Materials and methods:** A three-group community-based randomized controlled trial compared the effects of aerobic exercise, yoga and usual care (phone counselling) in physically inactive, non-treatment seeking, adults with AUD. Participants were recruited via advertisements. Assessments included Timeline Follow-back, AUD severity (DSM-5), Alcohol Use Disorders Identification Test (AUDIT) and biological markers. Primary outcome was weekly consumption at 13-weeks.

**Results:** 140 participants were randomized (mean age 54 years, SD = 12). More women (70%) than men were included. Participants drank on average 20 (SD = 11) standard drinks/week. Mean DSM-5 diagnostic criteria for AUD and AUDIT were 5 (SD = 2) and 17 (SD = 9) respectively. At follow-up, the within-group changes in consumption were statistically significant in all three groups: aerobic exercise mean = -5.0 (95% CI = -10.3, – 3.5), yoga mean = -6.9 (95% CI = -– 10.3, – 3.5) and TAU mean = -6.6 (95% CI = -– 8.8, – 4.4). No significant group differences were found on the primary or secondary outcomes. Per-protocol analyses favored yoga (mean = -– 8.7, 95% CI = -– 13.2, – 4.1) and usual care (mean = -– 7.1, 95% CI = -– 0.6, – 3.7) compared to aerobic exercise (mean = -– 1.7, 95% CI = -– 4.4, 1. 0), [F = 4.9, p = 0.011].

**Conclusions:** A 12-week exercise program has effects on alcohol consumption comparable to usual care. A per protocol analysis suggests that yoga tends to reduce consumption more than aerobic exercise.

